# A synthetic ion channel with anisotropic ligand response

**DOI:** 10.1038/s41467-020-16770-z

**Published:** 2020-06-10

**Authors:** Takahiro Muraoka, Daiki Noguchi, Rinshi S. Kasai, Kohei Sato, Ryo Sasaki, Kazuhito V. Tabata, Toru Ekimoto, Mitsunori Ikeguchi, Kiyoto Kamagata, Norihisa Hoshino, Hiroyuki Noji, Tomoyuki Akutagawa, Kazuaki Ichimura, Kazushi Kinbara

**Affiliations:** 1 School of Life Science and Technology, Tokyo Institute of Technology, 4259 Nagatsuta-cho, Midori-ku, Yokohama, 226-8503 Japan; 2 Precursory Research for Embryonic Science and Technology, Japan Science and Technology Agency, 4-1-8 Honcho, Kawaguchi, Saitama, 332-0012 Japan; 3 Institute of Multidisciplinary Research for Advanced Materials, Tohoku University, 2-1-1 Katahira, Aoba-ku, Sendai, 980-8577 Japan; 4 Institute for Frontier Life and Medical Sciences, Kyoto University, Shougoin, Kyoto, 606-8507 Japan; 5 Department of Applied Chemistry, School of Engineering, The University of Tokyo, Bunkyo-ku, Tokyo, 113-8656 Japan; 6 Graduate School of Medical Life Science, Yokohama City University, 1-7-29 Suehiro-cho, Tsurumi-ku, Yokohama, 230-0045 Japan; 7 Medical Sciences Innovation Hub Program RIKEN, 1-7-22 Suehiro-cho, Tsurumi-ku, Yokohama, 230-0045 Japan

**Keywords:** Membranes, Self-assembly

## Abstract

Biological membranes play pivotal roles in the cellular activities. Transmembrane proteins are the central molecules that conduct membrane-mediated biochemical functions such as signal transduction and substance transportation. Not only the molecular functions but also the supramolecular properties of the transmembrane proteins such as self-assembly, delocalization, orientation and signal response are essential for controlling cellular activities. Here we report anisotropic ligand responses of a synthetic multipass transmembrane ion channel. An unsymmetrical molecular structure allows for oriented insertion of the synthetic amphiphile to a bilayer by addition to a pre-formed membrane. Complexation with a ligand prompts ion transportation by forming a supramolecular channel, and removal of the ligand deactivates the transportation function. Biomimetic regulation of the synthetic channel by agonistic and antagonistic ligands is also demonstrated not only in an artificial membrane but also in a biological membrane of a living cell.

## Abstract (web summary)

Transmembrane proteins are important for cellular functions and synthetic analogues are of interest. Here the authors report on the design and testing of a synthetic multipass transmembrane channel which shows anisotropic responses to agonistic and antagonistic ligands.

## Introduction

Cellular and organelle membranes play pivotal roles in controlling and maintaining biological activities, including environmental sensing, energy conversion, signal transduction and substance transportation. A significant part of these functions is realized by a series of proteins so-called transmembrane proteins, and their structure–function relationships have been attracting interest not only in biology and medicine, but also in chemistry and materials science for developing functional molecules and nanodevices^1–3^. Inspired by the proteinic channels, for example, numerous types of synthetic supramolecular ion channels have been developed^4–6^, and ion transportation through biological membranes have been demonstrated^7,8^. On the other hand, it has also been recognized that not only the structure of the proteins itself, but also other features such as self-assembly, delocalization and orientation of the molecules in the membrane as well as their dynamic properties like stimuli-responsiveness, are also responsible for their functions. In this context, control of these features for synthetic molecules remains important challenges, and draws increasing interest to develop sophisticated stimuli-responsive systems like ligand-, light-, voltage- and tension-gated ion channels^9–11^. Indeed, only a few successful examples to control the orientation of the synthetic transmembrane molecules have been demonstrated, which critically limits the applicability of the synthetic molecules to sensing and separation devices^12,13^.

In this study, we report a totally synthetic multipass transmembrane channel that can be introduced in lipid bilayers unidirectionally, and shows anisotropic responses to ligands allowing reversible regulation of ion transportation through lipid bilayers. This synthetic transmembrane molecule shows agonistic and antagonistic responses to different ligands, and functions not only in an artificial membrane but also in a plasma membrane of a living cell.

## Results

### Molecular design and synthesis

**Fig. 1:**
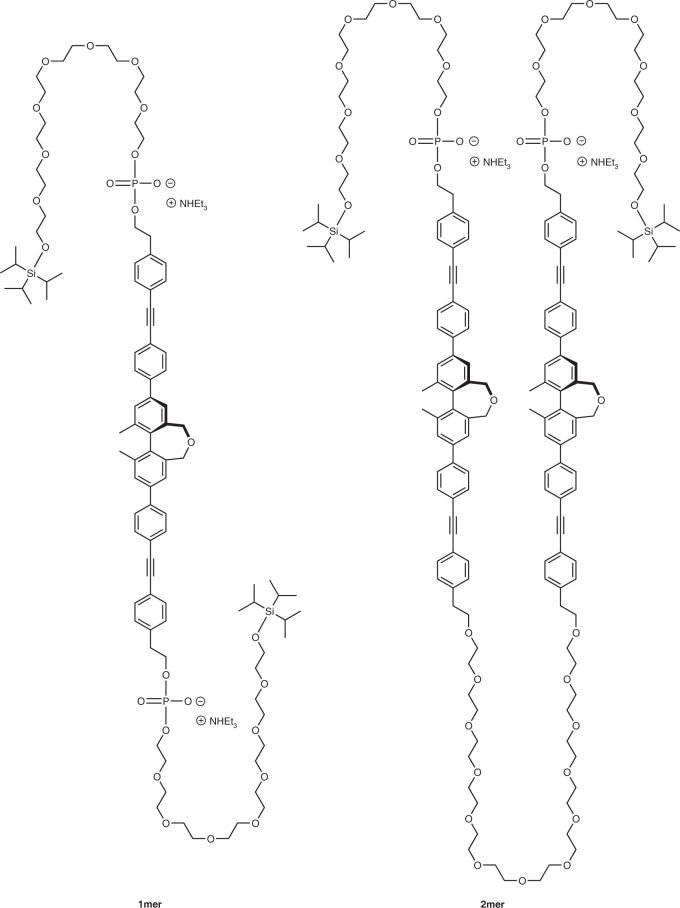
Molecular structures of multiblock amphiphiles. Molecular structures of **1mer** and **2mer**.

By mimicking a multipass transmembrane (MTM) structure seen in the proteinic ion channels constructed through folding of iterative hydrophilic and hydrophobic domains, we newly designed amphiphiles **1mer** and **2mer** (Fig. 1). These molecules have a multiblock structure with one and two hydrophobic (*R*)-1,11-dimethyl-3,9-bis[4-(phenylethynyl)phenyl]-5,7-dihydrodibenzo[*c*,*e*]oxepine (BPO) units and hydrophilic oligoethylene glycol (oligoEG) chains, where each terminus is capped with a hydrophobic triisopropylsilyl (TIPS) group, respectively (Fig. 1). While BPO unit and two oligoEG chains are connected via phosphate groups in the case of **1mer**, those of **2mer** are connected directly at one side and via a phosphate group at the other sides. These phosphate groups are expected to allow **1mer** and **2mer** to be dispersible to water and also to increase the interaction with aromatic amines as ligands. Furthermore, the BPO unit includes fluorescent and chiral components, which visualize the localization, conformational changes and assembling of these amphiphiles in the bilayer membranes through fluorescence and circular dichroism (CD) spectral measurements and microscopic observations. In addition, our previous study suggests that the TIPS groups at the termini of the oligoEG chains encourage the ion channel formation^7^.

**1mer** and **2mer** were prepared by coupling between BPO or BPO-dodecaEG-BPO and octaEG bearing TIPS and phosphite groups, followed by oxidation, respectively. BPO unit was synthesized by Suzuki-Miyaura cross coupling reaction between 2-(4-{[4-(4,4,5,5-tetramethyl-1,3,2-dioxaborolan-2-yl)phenyl]ethynyl}phenyl)ethan-1-ol and (*R*)-3,9-diiodo-1,11-dimethyl-5,7-dihydrodibenzo[*c*,*e*]oxepine, where the absolute configuration at the oxepine group was unambiguously determined by X-ray crystallographic analysis of its synthetic precursor bearing a chiral authentic group (Supplementary Methods, Supplementary Figs. 1–3).

### Conformations and self-assembling properties in solution

**Fig. 2:**
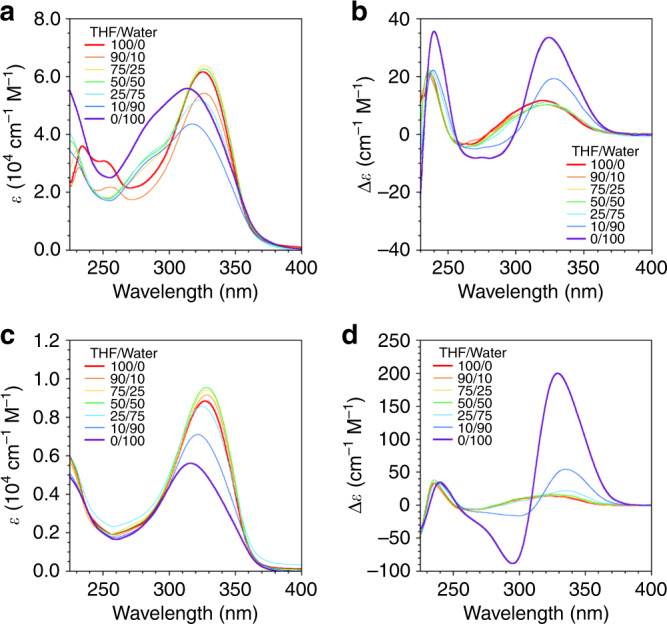
Spectroscopic characterization of self-assembly of multiblock amphiphiles upon solvent polarity change. **a**, **c** UV-vis absorption and **b**, **d** CD spectra of **a**, **b** **1mer** and **c**, **d** **2mer** in the mixture of THF and water at 20 °C. [**1mer**] = 14 μM. [**2mer**] = 7.0 μM.

Conformations and self-assembling properties of **1mer** and **2mer** were investigated by spectroscopic and light scattering measurements. Dynamic light scattering (DLS) measurements indicated that **1mer** was dispersed in tetrahydrofuran (THF) and formed aggregates in water (Supplementary Fig. 4a). **1mer** displayed blue shift of the absorption band with an emergence of a characteristic split Cotton effect upon increasing the ratio of water in THF, suggesting intermolecular H-type assembly of BPO units (*λ*_abs_ = 325 nm in THF, 314 nm in water, Fig. 2a, b). The fluorescence band of **1mer** showed a red-shift with a decrement of the intensity upon increase of the solvent polarity (*λ*_em_ = 383 nm in THF and 444 nm in water corresponding to the monomer and excimer emissions, respectively; fluorescence lifetime *τ*_383_ = 0.54 ns, *τ*_444_ = 2.13 ns, Supplementary Fig. 5).

Analogous to **1mer**, **2mer** forms aggregates in water (Supplementary Fig. 4b). This aggregation formation induced a blue-shift of the absorption band and significant CD spectral change to show a strong split Cotton effect (*λ*_abs_ = 329 nm and 316 nm in THF and water, respectively, Fig. 2c, d). Importantly, this CD profile in water was hardly dependent on the concentration of **2mer**. Namely, similar CD patterns were observed at concentrations of **2mer** in water of 7.0 μM and 2.5 mM (Supplementary Fig. 6). This similarity suggests that the CD signals in water are mostly originated from the geometry of the two aromatic units within a single molecule, thereby indicating an intramolecular assembly of BPO units of **2mer** in an aqueous medium. The overall fluorescence intensity of **2mer** decreased with an emergence of excimer emission upon addition of water (*λ*_em_ = 384 nm, *τ*_384_ = 0.15 ns in THF, *λ*_em_ = 411 nm, *τ*_411_ = 1.31 ns in water; Supplementary Figs. 5 and 7). Thus, it is likely that **2mer** adopts a folded conformation in water, where the two BPO units are in close proximity with each other, and form H-type assembly. ^1^H NMR spectroscopic measurements of **2mer** at 2.5 mM also support the folding in the aqueous media, where the aromatic proton signals showed an upfield shift upon addition of D_2_O to THF-*d*_8_, and DOSY measurements indicated that the observed ^1^H NMR signals correspond to the dispersed molecules of **2mer** (Supplementary Fig. 8, Supplementary Table 1).

### Ligand binding in solution

**Fig. 3:**
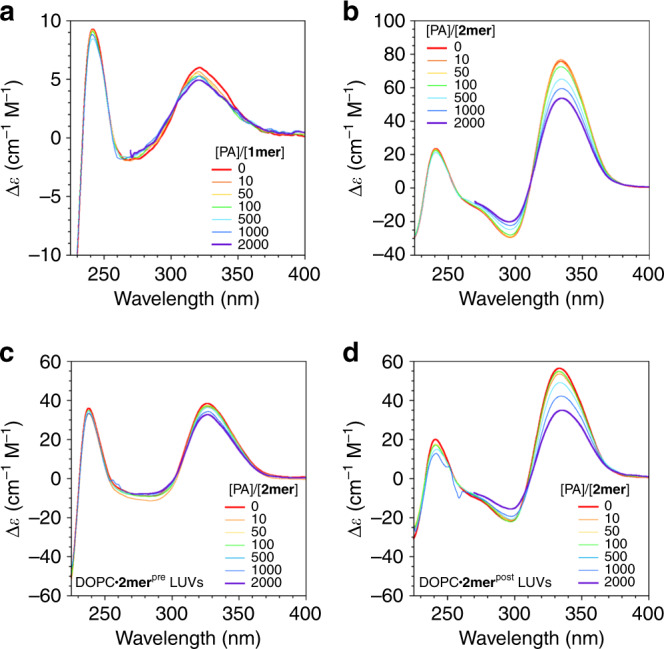
Characterization of ligand-binding capabilities of multiblock amphiphiles. CD spectra of **a** **1mer**, **b** **2mer**, **c** DOPC•**2mer**^pre^ LUVs and **d** DOPC•**2mer**^post^ LUVs in HEPES buffer at 20 °C upon titration with PA at [PA]/[**1mer**] = [PA]/[**2mer**] = 0.0, 10, 50, 100, 500, 1000 and 2000. [**1mer**] = 14 μM. [**2mer**] = 7.0 μM. Partial spectral curves at [PA]/[**1mer**] = [PA]/[**2mer**] = 2000 (*λ* < 270 nm) are eliminated due to unreliable signals caused by the strong absorption by PA. 20 mM HEPES, 50 mM KCl, 2.0 mM MgCl_2_, pH 7.5.

The folded conformation of **2mer**, with intramolecularly-stacked aromatic units with neighboring phosphate groups, was considered to be advantageous to binding with amine-type ligands bearing aromatic groups by electrostatic and aromatic-aromatic interactions, as found in the β-adrenergic receptor^14^. Thus, as simple ligands, 2-phenylethylamine (PA) and (*R*)-1-(isopropylamino)-3-(1-naphthyloxy)-2-propanol (propranolol, PPN) were chosen in this study. Upon addition of PA, **2mer** in a HEPES buffer showed continuous decrease in the intensity of CD signals (Fig. 3b). In ^1^H NMR spectra, the signals corresponding to the aromatic and aliphatic protons close to the phosphate groups showed upfield shifts in response to the PA addition (Supplementary Fig. 9). These spectral changes indicate that **2mer** binds with PA in an aqueous medium presumably at the connecting points of the phosphate and aromatic units by the electrostatic and aromatic interactions. Job’s plot indicates 1:1 complexation between **2mer** and PA, and the association constant was evaluated to be *K*_assoc_ = 1.07 × 10^2^ M^–1^ (Supplementary Figs. 10 and 11). Since the intensities of split CD signals depend on the distance and dihedral angle between two chromophores, the observed CD spectral change suggests a conformational change of **2mer** in company with the ligand accommodation. Essentially, similar spectral changes were observed in the titration of **2mer** with PPN, where the evaluated association constant was *K*_assoc_ = 1.04 × 10^4^ M^–1^ (*R* = 0.997, Supplementary Figs. 12 and 13). The stronger interaction of **2mer** with PPN than PA suggests a significant contribution of the aromatic-aromatic interaction to the formation of the complex. In contrast to **2mer**, titration of **1mer** with PA showed only little spectral changes (Fig. 3a, Supplementary Fig. 14). Thus, the folded conformation of **2mer** likely be essential for the ligand-binding capability of this amphiphilic receptor.

### Anisotropic membrane insertion and ligand binding

Having the folding and ligand-binding behaviours of **1mer** and **2mer** in hand, we investigated their incorporation as well as functions in liposomal membranes using giant and large unilamellar vesicles (GUVs, LUVs) prepared by the gentle hydration method. For the preparation of GUVs consisting of a mixture of 1,2-dioleoyl-*sn*-glycero-3-phosphocholine (DOPC) and **1mer**, we conducted two procedures, which we called preloading and postloading methods. Interestingly, these two methods unexpectedly gave contrasting results. In the preloading method, DOPC and **1mer** were dissolved in a mixture of MeOH and CHCl_3_. After evaporation of the resulting organic solution on the glass substrate, the residual film was hydrated with an aqueous solution of glucose and sucrose. Phase-contrast microscopy visualized successful formation of the GUVs (Supplementary Fig. 15a). Under fluorescence microscopic observation by irradiation with 330–385 nm light to excite the BPO units, ring-shaped images corresponding to the GUVs were visualized, indicating the localization of **1mer** in the DOPC liposomal membranes (Supplementary Fig. 15b). Detailed analysis by fluorescence depth quenching suggested a rather vertical orientation of the BPO units (Supplementary Fig. 16) in the membranes^15^. On the other hand, in the postloading method, **1mer** was added to the preformed DOPC GUVs in a buffer, which resulted in very faint fluorescent ring-shaped images of the GUVs with rather strong background fluorescence under fluorescence microscopic observation (Supplementary Fig. 15c, d). This suggests dispersion of **1mer** in the extravesicular aqueous medium. In the case of **2mer**, the preloading method also produced DOPC GUVs containing **2mer** localized in the bilayer (Supplementary Fig. 17a, b). Interestingly, in sharp contrast to **1mer**, **2mer** could be localized in the DOPC GUV membranes also by the postloading method (Supplementary Fig. 17c, d). Fluorescence depth quenching studies indicated a rather vertical orientation of the BPO units of **2mer** in the DOPC membranes prepared by both preloading and postloading methods (Supplementary Fig. 18). The CD and emission spectral profiles of **2mer** in the liposomal membranes show the characteristic split Cotton effect at 334 and 297 nm and excimer emissions at 387 and 408 nm (Fig. 3c, d red lines, Supplementary Figs. 19 and 20), indicating that **2mer** adopts the folded conformation in the membranes. These results suggest that the phosphate groups attached to the BPO units interfere with these molecules to pass through the DOPC membranes. Namely, while **1mer**, bearing the phosphate groups at the both ends, could not be inserted in the membrane by the postloading method, the folded form of **2mer** caused the unsymmetrical structure with the two phosphate groups locating at one side of the BPO units, likely allowing this molecule to be inserted into the membrane from the uncharged oligoEG chain side.

This biased insertion of folded **2mer** in the postloading method is expected to allow directed alignment of **2mer** in the DOPC membranes. Indeed, DOPC•**2mer** LUVs prepared by the preloading and postloading methods (DOPC•**2mer**^pre^ and DOPC•**2mer**^post^, respectively) showed significantly different ligand responses from each other. DOPC•**2mer**^pre^ LUVs showed a split Cotton effect at the wavelengths corresponding to the absorption of the BPO units, suggesting the folding of **2mer** in the DOPC bilayer (Fig. 3c, red line). Upon addition of PA, DOPC•**2mer**^pre^ LUVs showed only slight change in the CD spectra (Fig. 3c, Supplementary Fig. 19). In contrast, addition of PA to the extravesicular medium of DOPC•**2mer**^post^ LUVs caused more prompt change in the CD profile (Fig. 3d, Supplementary Fig. 20). Importantly, addition of **2mer** to the preformed DOPC LUVs, encapsulating PA in their internal aqueous phases, showed only small spectral difference from the DOPC•**2mer**^post^ LUVs without PA (Supplementary Fig. 21). These processing-dependent ligand-binding properties of **2mer** strongly indicate that the orientation of **2mer** in the bilayer is different in the liposomes prepared by the preloading and postloading methods, where the postloading method allows for anisotropic membrane insertion of **2mer** into the DOPC bilayer that exposes the phosphate groups to the extravesicular medium. The orientation selectivity of **2mer** in the DOPC bilayer prepared by the postloading method was estimated to be 95% by zeta potential measurements (Supplementary Fig. 22).

### Ligand-gated ion transportation

**Fig. 4:**
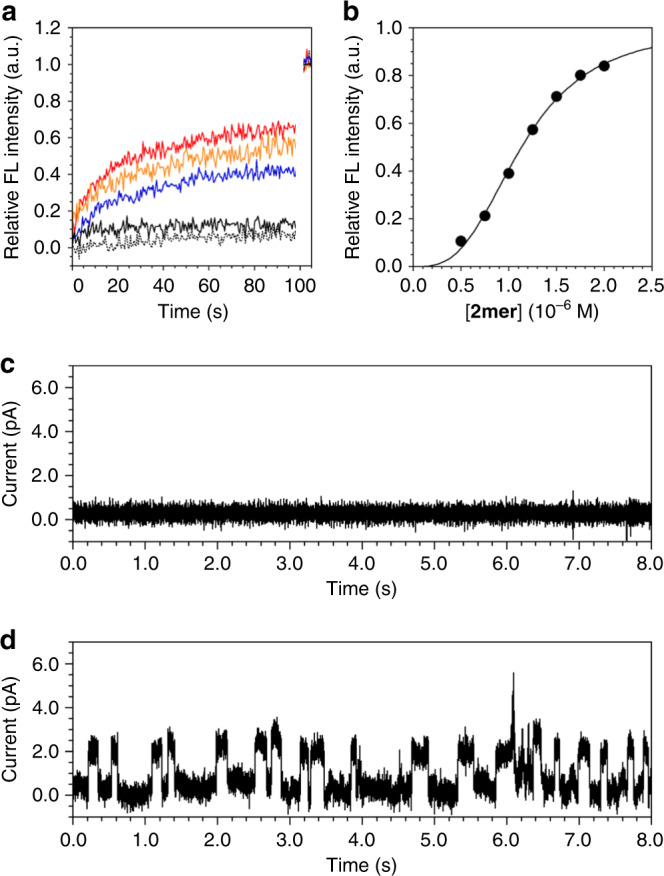
Ligand-gated ion transportation of membrane-embedded multiblock amphiphile. **a** Changes in the 510-nm fluorescence intensity of HPTS encapsulated in DOPC•**2mer**^post^ LUVs in HEPES buffer containing PA at 20 °C as a function of time after the addition of (red) LiOH, (orange) NaOH and (blue) KOH at 0 sec followed by addition of 1.0 wt% Triton X-100 at 100 sec ([DOPC] = 400 μM, [**2mer**] = 0.75 μM, [PA] = 10 mM, [HPTS] = 30 μM, 20 mM HEPES, 50 mM KCl, pH 7.1, excitation at *λ* = 460 nm, emission at *λ* = 510 nm). The black broken and solid lines show changes in the fluorescence intensities of HPTS encapsulated in (broken) DOPC•**2mer**^post^ LUVs and (solid) DOPC•**2mer**^post^ LUVs containing PA in the intravesicular medium in HEPES buffer in the absence of PA in the extravesicular medium upon addition of LiOH. ΔpH = 0.8 (7.1–7.9). **b** Plots of relative 510-nm fluorescence intensities of HPTS encapsulated in DOPC•**2mer**^post^ LUVs at 20 s after the addition of KOH in 20 mM HEPES buffer containing 50 mM KCl at 20 °C as a function of the concentration of **2mer** ([DOPC] = 400 μM, [PA] = 10 mM, [HPTS] = 30 μM). Curve-fitting analyses were carried out by the Hill equation. **c**, **d** Current traces at the applied voltage of 100 mV of a DOPC•**2mer**^post^ BLM ([DOPC]/[**2mer**] = 60,000/1) in HEPES buffer (20 mM HEPES, 50 mM KCl, 2.0 mM MgCl_2_, pH 7.5) **c** before and **d** after addition of PA (200 nM) at 20 °C.

Our previous studies demonstrated that the multipass transmembrane structures like **2mer**, consisting of alternatingly connected oligoethylene glycol chains and aromatic components are effective for ion transportation through formation of the supramolecular ion channels^9,11,16^. Based on the conformational change of **2mer** upon the ligand-binding, ligand-triggered ion transportation switching is also expected. Ion transportation capability of **2mer** was investigated using DOPC•**2mer**^post^ LUVs encapsulating 8-hydroxypyrene-1,3,6-trisulfonate (HPTS) in the internal aqueous phase, prepared in 20 mM HEPES buffer containing 50 mM KCl at pH 7.1 (DOPC•**2mer**^post^ LUV⊃HPTS, [**2mer**]/[DOPC] = 0.025). HPTS shows 510-nm fluorescence band upon 450-nm excitation at pH above 5, where the fluorescence intensity increases by increasing pH. In the absence of PA, DOPC•**2mer**^post^ LUV⊃HPTS hardly showed increment of the HPTS fluorescence intensity upon addition of LiOH (Fig. 4a, black broken line). In sharp contrast, in the presence of PA in the extravesicular medium of DOPC•**2mer**^post^ LUV⊃HPTS, elevation of the HPTS fluorescence intensity was readily observed upon addition of LiOH (Fig. 4a, red line), which visualizes the transportation of Li^+^ into the vesicles to raise the intravesicular pH. Importantly, DOPC•**2mer**^post^ LUV⊃HPTS encapsulating PA in the intravesicular medium hardly showed increase in the HPTS fluorescence intensity upon addition of LiOH to the extravesicular medium (Fig. 4a, black solid line). Thus, owing to the directed alignment of **2mer** in the bilayer membrane, anisotropic regulation of the ligand-gated ion transportation could be realized. The HPTS assay of DOPC•**2mer**^post^ LUV⊃HPTS shows the fastest permeation of Li^+^ followed by Na^+^ and K^+^ (Fig. 4a).

The ion transportation capability of **2mer** with its anisotropic ligand response was further investigated using a DOPC black lipid membrane (BLM) for real-time single-channel current recording. The DOPC BLM was formed horizontally at the orifice by painting the *n*-decane solution of the lipid. The orifice was sandwiched by two chambers (upper and lower chambers) containing an electrolyte buffer solution. After formation of the DOPC BLM, we added **2mer** to the upper chamber expecting its insertion into the DOPC bilayer with directed alignment (DOPC•**2mer**^post^ BLM, [DOPC]/[**2mer**] = 60,000/1). Currents were recorded as a function of time at 20 °C, where DOPC•**2mer**^post^ BLM before addition of a ligand hardly showed current signals (Fig. 4c). Addition of PA (200 nM) into the *upper* chamber of the DOPC•**2mer**^post^ BLM readily triggered a current flow, where the square-top-shaped currents with average flows of 1.4 pA were observed at 100 mV, likely corresponding to ion transportation through a singly-formed ion channel (Fig. 4d). In contrast, addition of PA into the lower chamber of the DOPC•**2mer**^post^ BLM hardly induced the intensified current signals, while the subsequent addition of PA into the upper chamber triggered the ion transportation (Supplementary Fig. 23). As expected, BLM prepared from *n*-decane solution of a mixture of DOPC and **2mer** (DOPC•**2mer**^pre^ BLM) responded to the PA addition to both upper and lower chambers identically (Supplementary Fig. 24). Thus, the ion transportation of **2mer** was controlled by the ligand complexation, where addition of **2mer** to the preformed BLM allowed for the anisotropic response to the ligand. The relationship between the inner diameter *d* and the conductance *g* of an ion channel (1.4 pS) is known to be described by the Hille equation,

1 $$\frac{1}{g} = \frac{{l\rho }}{{\pi \left( {d/2} \right)^2}} + \frac{\rho }{d}$$

where *l* and *ρ* are the length of the channel (3.5 nm) and resistivity of the recording solution (2.35 Ω m), respectively^17^. By solving the equation including the Sansom correction factor, *d* was estimated to be 0.39 nm^18^. 100-Times dilution of PA in the upper chamber of the DOPC•**2mer**^post^ BLM resulted in a silent current profile and the signals recovered after the second addition of PA to the upper chamber (Supplementary Fig. 25), suggesting reversible ligand-gated response of **2mer** to PA. Hill analysis on the **2mer** concentration dependency of the cation transportation rate in the HPTS assay indicated the Hill coefficient *n* = 3.06 (*R* = 0.998), suggesting supramolecular ion channel formation of **2mer**, in which the number of **2mer** molecules constructing the ion channel is likely multiple of three (Fig. 4b)^19,20^. Among the alkali metal cations, Li^+^ was transported in the fastest rate. **2mer** transported alkaline-earth metal cations such as Ca^2+^ as well (Supplementary Fig. 26).

It should be noted here that, PPN, known as an antagonist for the β-adrenergic receptor, showed a contrastive effect to PA on the ion transportation of **2mer**. In fact, addition of PPN (50 nM) to the upper chamber of DOPC•**2mer**^post^ BLM hardly prompted current flow, and even subsequent addition of PA (200 nM) did not trigger the current flow (Supplementary Fig. 27a, b). Furthermore, subsequent addition of PPN to the upper chamber of the DOPC•**2mer**^post^ BLM, after addition of **2mer** in the presence of PA, deactivated the current flow (Supplementary Fig. 27c, d). Thus, analogous to the agonistic and antagonistic effects of PA and PPN to β-adrenergic receptor, PA and PPN apparently act to **2mer** like an agonist and an antagonist, respectively. NMR and modelling studies of **2mer**–ligand complexes indicated that PA and PPN bind with **2mer** through different interacting modes (Supplementary Figs. 28 and 29). Namely, electrostatic interaction between the phosphate and ammonium groups is likely dominant between PA and **2mer**, and PA is located at the rim of the channel. Meanwhile, hydrophobic interaction is preferred between **2mer** and PPN, therefore PPN is inserted into the hydrophobic cavity of the channel to infill the pore.

### Regulation of ion transportation through biological membrane

**Fig. 5:**
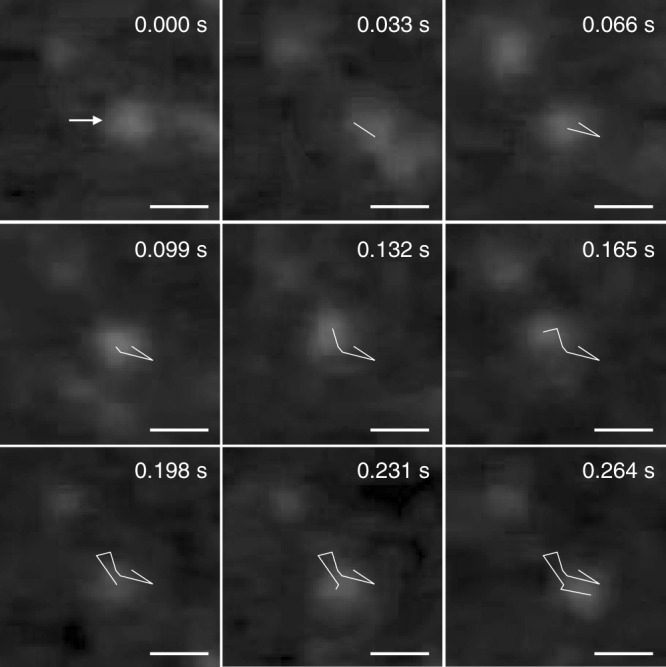
Localization of multiblock amphiphile in plasma membrane. Snapshots of total internal reflection fluorescence microscopic observation of **Cy3-2mer** in the plasma membrane of mouse L cell at an interval of 0.033 s at 25 °C. White lines represent the track of lateral migration of the fluorescence spot pointed by a white arrow. Scale bars: 1.0 μm.

**Fig. 6:**
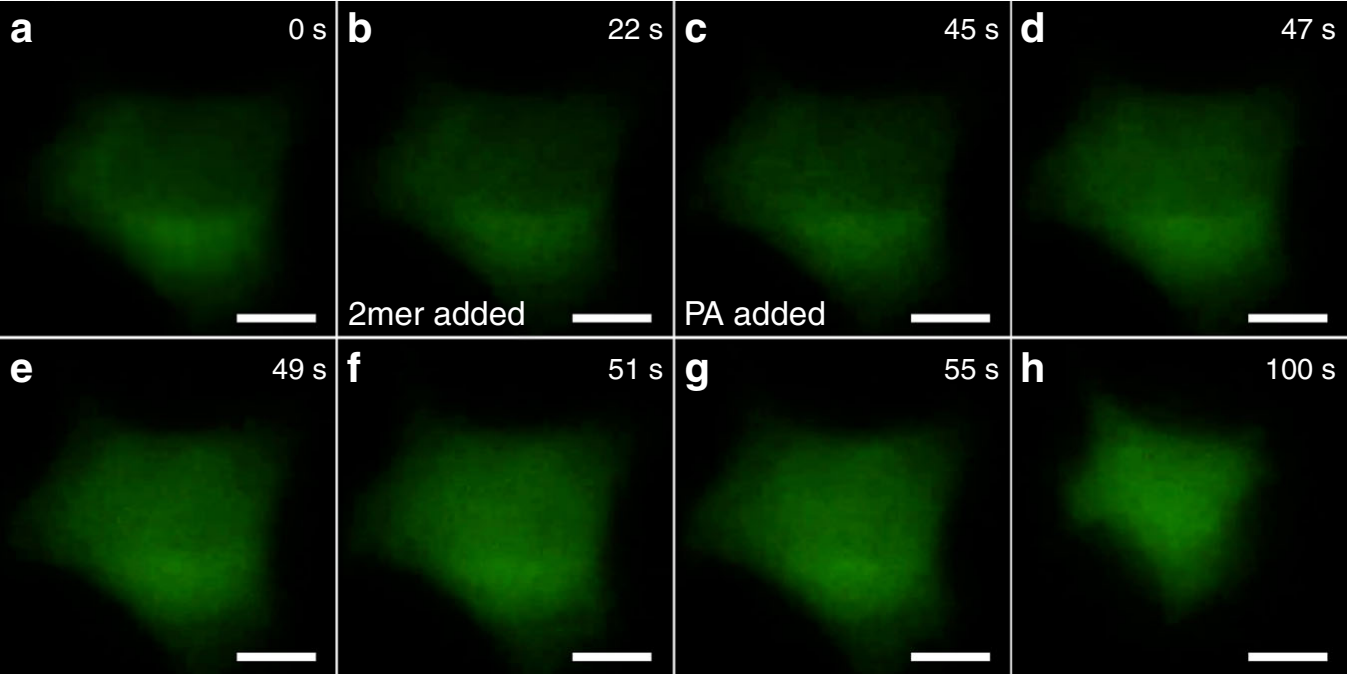
Ligand-gated ion transportation of multiblock amphiphile embedded in plasma membrane. Snapshots of fluorescence microscopic observation of L cells encapsulating Fluo-4 at 25 °C at **a** 0 s (at the beginning of the observation), **b** 22 s (after the addition of **2mer**) and **c**–**h** 45–100 s (after the addition of PA). Scale bars: 5.0 μm.

Taking advantage of the capability of **2mer** to spontaneously insert into the membrane, with its ligand-gated ion transportation function, we further challenged the regulation of ion transportation through a plasma membrane of a living cell. For visualization of **2mer** in the cell culture by fluorescence microscopy, cyanine3-labelled **2mer** (**Cy3-2mer**) was prepared. The localization of **Cy3-2mer** in the plasma membrane was investigated by total internal reflection fluorescence (TIRF) microscopic observation with 561-nm laser excitation to visualize Cy3 fluorescence. TIRF microscopy is advantageous to observation of molecules embedded in a live-cell plasma membrane because of its high sensitivity to visualize fluorescent species near the coverslip-specimen interface. **Cy3-2mer** was added to mouse L cells cultured in HBSS buffer (final concentration of **Cy3-2mer** is 10 nM), where the TIRF microscopy displayed multiple fluorescent spots corresponding to the fluorescence from a single molecule of **Cy3-2mer**. Importantly, fluorescent spots showing lateral diffusion were observed within a domain the cell exists, suggesting the localization of **Cy3-2mer** in the plasma membrane (Fig. 5, Supplementary Fig. 30, Supplementary Movie 1). It should be noted here that addition of the Cy3 fragment alone to the cells hardly showed such lateral migration of the fluorescent spots, while flashing spots were dominantly observed (Supplementary Movie 2). Thus, it is strongly likely that the multiblock amphiphile **Cy3-2mer** could be inserted into the plasma membrane of L cells. For the assay of the ligand-gated ion transportation in the biological environment, we prepared L cells encapsulating a green-fluorescent calcium-ion indicator, Fluo-4, to visualize Ca^2+^ flow into a cell. Under a fluorescent microscopic observation with 488-nm excitation, L cells encapsulating Fluo-4 showed weak background green fluorescence (Fig. 6a), where addition of ionomycin, an ionophore known to transport Ca^2+^, resulted in enhancement of the fluorescence intensity of Fluo-4 (Supplementary Fig. 31). Meanwhile, since L cells do not possess β-adrenergic receptors, addition of PA to L cells without **2mer** did not prompt the apparent change in the fluorescence intensity of Fluo-4 (Supplementary Fig. 32). In sharp contrast, while addition of **2mer** to the cells hardly changed the fluorescence intensity ([**2mer**] = 10 nM, Fig. 6b and Supplementary Fig. 33), subsequent addition of PA readily prompted significant enhancement of the Fluo-4 fluorescence intensity in the cells (Fig. 6c–h, Supplementary Figs. 34 and 35, Supplementary Movie 3, [PA] = 10 μM). Importantly, enhancement of the Fluo-4 fluorescence intensity was not observed by addition of PPN to L cells, which remained unchanged even by subsequent addition of PA ([PPN] = 100 nM, Supplementary Fig. 36).

In summary, we demonstrate anisotropic ligand responses of a synthetic multipass transmembrane ion channel. An unsymmetrical molecular structure enables oriented insertion of the synthetic amphiphile to a bilayer by addition to a preformed membrane. Complexation with a ligand prompts formation of a supramolecular channel for ion transportation, and removal of the ligand deactivates the transportation function. Biomimetic regulation of the supramolecular channel by agonistic and antagonistic ligands is also demonstrated not only in an artificial membrane but also in a biological membrane of a living cell.

## Methods

### Materials

Triton X-100 was purchased from Alfa Aesar. DOPC and Doxyl PCs were purchased from Avanti Polar Lipids. Deuterated solvents were purchased from Kanto Chemicals. Anhydrous Na_2_SO_4_, CHCl_3_, CuI, glucose, HEPES, KCl, KOAc, KOH, MgCl_2_, NaCl, NH_4_Cl and tetrahydrofuran (THF) were purchased from Nacalai Tesque. Ag_2_CO_3_, [1,1’-bis(diphenylphosphino)ferrocene]dichloropalladium(II) complex with dichloromethane (Pd(dppf)Cl_2_•CH_2_Cl_2_) and dry NEt_3_ were purchased from Sigma–Aldrich. Bis(pinacolato)diboron, bis(triphenylphosphine)palladium(II) dichloride (Pd_2_Cl_2_(PPh_3_)_2_), 1-bromo-4-iodobenzene, *α*-cyano-4-hydroxycinnamic acid (CHCA), *n*-decane, 4-dimethylaminopyridine, 8-hydroxypyrene-1,3,6-trisulfonate (HPTS), I_2_, NaH, Na_2_S_2_O_3_, pivaloyl chloride, salicylchlorophosphite, triethylammonium bicarbonate, Pd(PPh_3_)_4_, quinine and quinidine were purchased from Tokyo Chemical Industry. Dry MeOH and dry pyridine were purchased from Wako Pure Chemical. These commercial reagents were used without purification. Dry CH_2_Cl_2_ and dry THF were purchased from Kanto Chemical and passed through sequential two drying columns on a Glass-Contour system just prior to use. Column chromatography was carried out with Silica Gel 60 (spherical, neutral, particle size: 63–210 μm) purchased from Kanto Chemical, Chromatorex-DIOL silica (spherical, pH 9.5, particle size: 100 μm) purchased from Fuji Silysia Chemical or bio-beads (S-X1 or S-X3) purchased from Bio-Rad. Deionized water (filtered through a 0.22 μm membrane filter, >18.2 MΩ cm) was purified in a Milli-Q system of Millipore Corp.

### Instrumentation

Nuclear magnetic resonance (NMR) spectra were recorded on a 400 MHz FT NMR Bruker BioSpin AVANCE III 400, where the chemical shifts were determined with respect to a solvent signal as an internal standard (^1^H NMR: 7.24 ppm for CDCl_3_, 2.05 ppm for acetone-*d*_6_; ^13^C NMR: 77.16 ppm for CDCl_3_, 28.98 ppm for acetone-*d*_6_) or a reference signal as an external standard (^31^P NMR: 0.00 ppm for 6% H_3_PO_4_ aq.). Matrix-assisted laser desorption/ionization time-of-flight mass spectrometry (MALDI-TOF MS) was performed in reflector mode with CHCA as a matrix on a Bruker Daltonics autoflex speed spectrometer. High-resolution electrospray ionization (HR ESI) TOF MS spectra were recorded on a Bruker micrOTOF-Q II-S1 with MeOH as a solvent. Analytical thin layer chromatography (TLC) was performed on precoated, glass-backed silica gel Merck 60 F254 or Fuji Silysia Chemical Chromatorex Diol TLC. Visualization of the developed chromatogram was performed by UV absorbance, Hanessian’s stain or iodine. Analytical high-performance liquid chromatography (HPLC) was performed with a system composed of JASCO PU-2080 Plus Intelligent HPLC Pump, UV-2077 Plus 4-*λ* Intelligent UV/VIS Detector, DG-2080-53 3-Line Degasser, LG-2080-02 Ternary Gradient Unit and LC-Net II/APC system. X-ray crystallographic analyses were carried out with Rigaku RAPID II refractometer. Fluorescent and phase-contrast microscopic observation was performed with an Olympus IX-71 microscope, where a U-MWU2 mirror unit (excitation filter: 330–385 nm, emission filter: 420 nm, dichroic mirror: 400 nm) was used for fluorescence observation. On a slide glass, a coverslip was placed over the object through a 0.1-mm thick silicon-based spacer. UV absorption spectra were recorded on JASCO V-530 UV-Vis spectrophotometer. Circular dichroism (CD) spectra were recorded on JASCO J-820 spectropolarimeter. Fluorescence spectra were recorded on JASCO FP-6500 spectrofluorometer. Fluorescence lifetime was measured with Hamamatsu Photonics Quantaurus-Tau C11367 fluorescence lifetime spectrometer. Dynamic light scattering (DLS) measurement was performed with a Malvern Zetasizer Nano ZS, where a Zetasizer nano series DTS 1060 Folded capillary cell or a 1-cm-thick quartz cell was used. Zeta potential measurement was performed with a Malvern Zetasizer Nano ZSP, where a Zetasizer nano series DTS 1070 Folded capillary cell was used.

### Giant unilamellar vesicles preparation

To a mixture of CHCl_3_/MeOH (2/1 v/v, 10 μL) in a glass test tube, were added a CHCl_3_ solution of DOPC (2.0 mM, 20 μL) and a MeOH solution of glucose (10 mM, 12 μL). The resulting mixture was gently dried under Ar flow to produce thin lipid film. The film was subsequently dried under vacuum over 3 h at 25 °C and hydrated overnight with 200 mM sucrose aq. (200 μL) under Ar at 37 °C. To the vesicle suspension was added an appropriate amount of **1mer** or **2mer** dispersed in HEPES buffer.

### Large unilamellar vesicles preparation

To a mixture of CHCl_3_/MeOH (2/1 v/v, 50 μL) put in a glass test tube, was added a CHCl_3_ solution of DOPC (2.0 mM, 100 μL). The resulting mixture was gently dried under Ar flow to produce thin lipid film, which was subsequently dried under vacuum over 3 h at 25 °C and hydrated overnight in HEPES buffer (20 mM HEPES, 50 mM KCl, 2.0 mM MgCl_2_, pH 7.5) under Ar at 37 °C. The resulting mixture was stirred on a shaker (203 min^–1^) for 1 h at 37 °C, followed by vortex mixing for 10 s, freezing-and-thawing for three times, and subsequent vortex mixing for 10 s. After being left standing overnight at 37 °C, the resulting mixture was passed through a polycarbonate membrane of 100-nm pore size (LFM-100) attached in a LiposoFast-Basic device by pushing the sample back and forth between the two gas-tight syringes over 11 times. To the vesicle suspension was added an appropriate amount of **1mer** or **2mer** dispersed in HEPES buffer.

LUVs for fluorescence depth quenching were prepared by following the above procedure using a mixture of DOPC and 5-Doxyl PC, 12-Doxyl PC, or 16-Doxyl PC ([DOPC]/[Doxyl PC] = 90/10).

LUVs encapsulating HPTS in the inner aqueous phase were prepared by following the above procedure, where 20 mM HEPES buffer containing 50 mM KCl (pH 7.1) and 30 μM HPTS was used as the hydration medium. After the extrusion process, the obtained suspension was dialyzed at 4 °C in 20 mM HEPES buffer containing 50 mM KCl (pH 7.1, 1.0 L, three times) using Spectra/Por Dialysis Membrane (MWCO 3500).

### Zeta potential measurement

DOPC•**2mer**^pre^ LUVs ([DOPC] = 10 mM, [**2mer**] = 500 μM) were prepared by following the above procedure using a mixture of DOPC and **2mer** in CHCl_3_ ([**2mer**]/[DOPC] = 1/20). To a stirred solution of 20 mM HEPES buffer containing 50 mM KCl and 2.0 mM MgCl_2_ (980 μL, pH 7.5) was added a HEPES buffer suspension of DOPC•**2mer**^pre^ LUVs ([DOPC] = 10 mM, [**2mer**] = 500 μM, 20 μL), and the mixture was further pipetted for five times.

DOPC LUVs ([DOPC] = 10 mM) were prepared by following the above procedure. To a stirred suspension of DOPC LUVs in 20 mM HEPES buffer containing 50 mM KCl and 2.0 mM MgCl_2_ (980 μL, pH 7.5) was added a HEPES buffer dispersion of **2mer** ([**2mer**] = 1 mM, 20 μL), and the mixture was further pipetted for five times.

Zeta potential was measured from the electrophoretic mobility using a Smoluchowski model, and obtained values were averaged from 10 independent runs. All zeta potential measurements were performed at 20 °C.

### Fluorescence measurement for ion transportation study

To a DOPC•**2mer** LUV⊃HPTS suspension ([DOPC] = 400 μM, [**2mer**] = 0.75 μM, [HPTS] = 30 μM) in 20 mM HEPES buffer containing 50 mM KCl (1.99 mL, pH 7.1) was added an aqueous solution of KOH (0.60 M, 10 μL, ΔpH = 0.8) by a syringe in the dark at 20 °C. Fluorescence intensity of HPTS at 510 nm upon excitation with 460 nm-light was monitored as a function of time until the addition of 1.0 wt% Triton X-100 (40 μL) at 100 s. Relative fluorescence intensity of HPTS in response to the pH enhancement was evaluated by the equation of

2 $$I = \frac{{I_{\mathrm{t}} - I_0}}{{I_{\mathrm{1yzed}} - I_0}}$$

where *I*_0_, *I*_t_ and *I*_lyzed_ represent the fluorescence intensities before addition of KOH, at *t* seconds after addition of KOH, and after lysis by the addition of 1.0 wt% Triton X-100, respectively.

### Conductance measurements using BLM system

A CHCl_3_ solution of DOPC (12.7 mM, 100 μL) was gently dried under N_2_ flow, which was then dispersed in *n*-decane (100 μL). The *n*-decane suspension (5 μL) of DOPC was painted on an orifice (diameter 150 μm) sandwiched by two chambers containing HEPES buffer (upper chamber: *trans*, lower chamber: *cis*, 20 mM HEPES, 50 mM KCl, 2.0 mM MgCl_2_, pH 7.5, 0.30 mL each) followed by the addition of an appropriate amount of **2mer** dispersed in HEPES buffer. Current was measured with a Nihon Kohden CEZ2400 amplifier and stored on a computer using an AD Instruments PowerLab at 40 kHz sampling rate. Recordings were filtered at 1 kHz. All the current recordings were performed at 20 °C.

### Cell culture

Mouse L cells, a kind gift from Dr. H. Fujisawa of Nagoya University, were cultured in Dulbecco’s Modified Eagle’s Medium (Sigma) supplemented with 10% (v/v) FBS, Penicillin G (100 units/mL) and Streptomycin sulfate (100 µg/mL, Wako) at 37 °C in 5% CO_2_. The cells were seeded in a glass-base dish (35-mm diameter with a window diameter of 12 mm, 0.12~0.17-mm thick glass; Iwaki) and cultured for at least 24 h before observation.

### Single-molecule fluorescence tracking by TIRF

Single fluorescent-molecule observation was performed as previously described^21^. To briefly explain, an objective lens-type TIRF (Total Internal Reflection fluorescence) microscope based on an Olympus IX-81 was employed. Mouse L cells were observed at room temperature of 25 °C after addition of **Cy3-2mer** (10 nM) in HBSS-HEPES buffer (HBSS supplemented with 10 mM HEPES, adjusted to pH 7.4 with NaOH). The bottom plasma membrane was locally illuminated with an evanescent field (an Olympus ×100, 1.49 NA objective lens; excitation at 561 nm). The fluorescent image of **Cy3-2mer** was projected onto a two-stage microchannel plate intensifier (C8600-03; Hamamatsu Photonics), and its output image was then lens coupled to an electron bombardment charge-coupled device camera (C7190-23; Hamamatsu Photonics). The obtained images were recorded at 30 Hz on a digital video deck for the following analysis. These instruments were also employed for fluorescence imaging of intracellular Ca^2+^ mobilization (described in the next section).

### Fluorescence microscopic observation of L cells

The mouse L cells were incubated with HBSS-HEPES buffer in the presence of Fluo-4-AM (4.6 μM), Probenecid (1.25 mM) and Pluronic F127 (0.04%) for 1 h at 37 °C, and washed three times with HBSS-HEPES buffer, and then fresh HBSS-HEPES buffer were added. During the observation, **2mer** (10 nM) was added, followed by addition of ionomycin (0.9 μM) as a positive control of Ca^2+^ mobilization. The cells were illuminated with laser light with a wavelength of 488 nm, and observed at room temperature with the same Olympus IX-81.

### Modelling of 2mer and ligand complexes

A three dimensional structure of a **2mer** was built by 2D Sketcher and Minimize-Selected-Atoms modules in MAESTRO (Shrödinger release 2018-2). The silicon atom at the TIPS group was replaced with a carbon atom. The folded structure of the **2mer** was set to the M-shape as shown in Fig. 1. After building the one **2mer** structure, other two **2mer** structures were further built, and a three-**2mer** complex was prepared, because it was suggested that the supramolecular ion channel formation of **2mer** estimated by the Hille equation was achieved by multiple of three. The initial formation of the three-**2mer** complex was set to a symmetric triangle form, and the directions of the three **2mer** were aligned (Supplementary Fig. 29a). The three dimensional structures of PA and PPN were built by the same protocol for **2mer**. A ligand was located between the two BPO units for one **2mer**, and the three ligands were arranged one-to-one with the three **2mer**. The position of ligand was located at the position of benzene near the phosphorus atom in **2mer**. To reduce steric hindrances among TIPS groups for each **2mer** or among **2mer** and PA, the structures around the phosphate part near PA and the positions of the TIPS group were modified manually. Using the modified structure of the three-**2mer** complex, each PA was replaced with PPN, and these positions of PA (Supplementary Fig. 29b) or PPN (Supplementary Fig. 29c) were matched as possible.

The three-**2mer** and ligand complex were embedded in DOPC membrane and water molecules using the Membrane Builder implemented in CHARMM-GUI^22–27^. The orientation of **2mer** relative to a lipid bilayer was set vertically, and the buried area of **2mer** was determined so that the phosphate part of **2mer** and DOPC molecules were close to each other. The molecular dynamics unit cell was set to a rectangular cell. In the center of the cell, the complex was embedded in a 70 Å × 70 Å DOPC bilayer at the x–y plane, and the number of DOPC molecules at the upper and lower leaflets was 56 and 65, respectively. Along the *z*-axis of the unit cell, water molecules were added, and the water thickness was set to 22.5 Å. Counterions and 150 mM KCl were included. A three-**2mer** and PA complex embedded in DOPC membrane and water molecules is illustrated in Supplementary Fig. 29d.

The complex embedded in the membrane-water system was equilibrated by all-atom molecular dynamics (MD) simulations. MD simulations were performed using the MD program package GROMACS ver. 2016.3^28–30^ under periodic boundary conditions. The CHARMM36m force field was used for membranes^31–35^ and the TIP3P water model^36^, and the CHARMM General Force Field (CGenFF)^37^ was used for **2mer** and ligands. The CGenFF parameters were assigned by ParamChem through the Membrane Builder. For CGenFF parameters of **2mer**, the parameters for each part of the oligoEG including the TIPS group, the BPO unit, the PEG were assigned piece by piece, and then these parameters were assembled. The electrostatic interaction was handled by the smooth particle mesh Ewald method^38^, and the van del Waals interaction was truncated by the switching function with the range of 10–12 Å. Bond lengths involving hydrogen atoms were constrained by the P-LINKS algorithm^39^. The temperature and pressure were 300 K and 1 atm, respectively. According to the default setup in the Membrane Builder, an energy minimization by the steepest descent method and six equilibration simulations, written as EQ1-EQ6 hereafter, were performed sequentially before the production run. The ensemble adopted in the simulations was NVT ensemble in EQ1 and EQ2 and NPT ensemble in EQ3 to EQ6 and the production run. The thermostat in the equilibration runs was the weak-coupling scheme of Berendsen^40^, and that in the production run was the Nosé–Hoover scheme^41,42^. The barostat in EQ3 to EQ6 was the semi-isotropic Berendsen algorithm^40^, and that in the production run was the semi-isotropic Parrinello–Rahman approach^43,44^. The time step was set to 1 fs in EQ1 to EQ3, and 2 fs in EQ4 to EQ6 and the production runs. The simulation length of each simulation was 125 ps for EQ1 to EQ3, 500 ps for EQ4 to EQ6, and 500 ps for a production run.

In the equilibration processes, structural restraints were imposed on **2mer**, ligand, and DOPC molecules according to the default setup of the Membrane Builder. The position harmonic restraints were imposed on heavy atoms of **2mer** and ligand. During the equilibration runs, the strength of the force constant was reduced gradually from 4000 to 50 kJ·mol^–2^·nm^–2^. In DOPC molecules, the *z*-axis of the phosphorus atom was restrained by the position harmonic restraints, and its force constant was gradually reduced as 1000–0 kJ·mol^–2^·nm^–2^. In addition, three dihedral angles were restrained. One was the dihedral angle among three carbon atoms at the *sn*-1, -2, -3 positions and an adjacent oxygen atom at the *sn*-2 position. The angle was restrained at 120 degree. The other was the two dihedral angles corresponding to the double bond between carbon atoms in the oleic acid part in the *sn*-2 and -3 positions, and the angle was restrained at the *cis* conformation. The force constants of these dihedral angles were reduced gradually from 1000 to 0 kJ·mol^–2^·rad^–2^.

After the equilibration runs, a 500 ps production run was carried out for each the three-**2mer** and PA complex and the three-**2mer** and PPN complex. In the production runs, no restraints were imposed.

### Reporting summary

Further information on research design is available in the Nature Research Reporting Summary linked to this article.

## Supplementary information

Supplementary Information

Description of Additional Supplementary Files

Supplementary Movie 1

Supplementary Movie 2

Supplementary Movie 3

Reporting Summary

## Data Availability

The authors declare that the data that support the findings of this study are available within the paper and its Supplementary Information file. All other information is available from the corresponding authors upon reasonable request.
