# Supplementary material for: A synthetic ion channel with anisotropic ligand response
Source: Nat Commun. 2020 Jun 10;11:2924. doi: 10.1038/s41467-020-16770-z (PMC7287108; doi:10.1038/s41467-020-16770-z)
Supplement: Supplementary file 1 — Supplementary Information [file 41467_2020_16770_MOESM1_ESM.pdf]

Supplementary Information

A synthetic ion channel with anisotropic ligand response

Muraoka *et al.*

Supplementary Methods

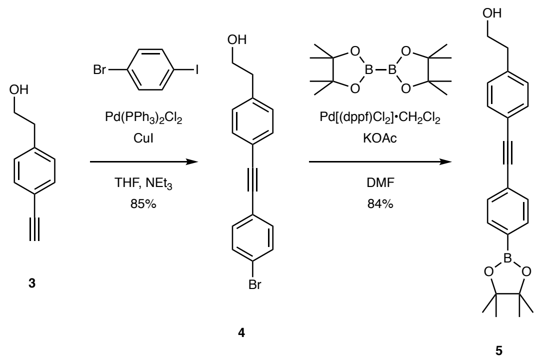

Synthesis of 4. To a dry NEt_3 (15 mL) solution of 1-bromo-4-iodobenzene (1.80 g, 6.36 mmol), $Pd_2Cl_2(PPh_3)_2$ (92.5 mg, 0.132 mmol) and CuI (41.9 mg, 0.220 mmol) were added **3** (855 mg, 5.85 mmol) in THF (15 mL) at 0 °C under Ar, and the resulting mixture was stirred for 6 h at 20 °C. Then, the reaction mixture was evaporated to dryness under reduced pressure, and the residue was chromatographed on silica gel (Silica Gel 60) with $CHCl_3$ as an eluent, followed by reprecipitation with $CHCl_3$ and hexane to allow isolation of **4** (1.50 g, 5.00 mmol) as colorless needle crystals in 85% yield.

TLC (Merck 60 F254, $CHCl_3$) R_f = 0.18; ^{1}H NMR (400 MHz, $CDCl_3$, 25 °C): δ = 7.46 (d, J = 8.0 Hz, 2H), 7.45 (d, J = 8.0 Hz, 2H), 7.36 (d, J = 8.0 Hz, 2H), 7.21 (d, J = 8.0 Hz, 2H), 3.86 (q, J = 11.2, 6.4 Hz, 2H), 2.87 (t, J = 6.4 Hz, 2H), 1.40 (brs, 1H) ppm; ^{13}C NMR (125 MHz, $CDCl_3$, 25 °C): δ = 139.45, 133.21, 132.00, 131.82, 129.36, 122.61, 122.49, 121.25, 90.59, 88.35, 63.62, 39.30 ppm; HR ESI-TOF MS (MeOH, positive mode): m/z = 301.0230 (calculated m/z on the basis of the monoisotropic mass of $C_{16}H_{13}BrNaO$ $[M + H]^+$ = 301.0228).

Synthesis of 5. To a degassed dry DMF (50 mL) solution of **4** (2.40 g, 7.97 mmol), bis(pinacolato)diboron (2.37 g, 9.33 mmol) and KOAc (2.66 g, 27.13 mmol) were added $Pd(dppf)Cl_2$•CH_2Cl_2 (289 mg, 35.4 μmol) at 20 °C under Ar. The reaction mixture was stirred for 4 h at 80 °C. Then, the mixture was evaporated to dryness under reduced pressure at 20 °C, and the residue was chromatographed on silica gel (Silica Gel 60) with a gradient from a mixture of $CHCl_3$ and hexane (1/2 v/v) to $CHCl_3$ as an eluent, followed by reprecipitation with $CHCl_3$ and hexane to allow isolation of **5** (2.35 g, 6.73 mmol) as colorless needle crystals in 84% yield.

TLC (Merck 60 F254, $CHCl_3$) R_f = 0.12; ^{1}H NMR (400 MHz, $CDCl_3$, 22 °C): δ = 7.76 (d, J = 8.0 Hz, 2H), 7.50 (dd, J = 8.0 Hz, 2H), 7.46 (d, J = 8.4 Hz, 2H), 7.20 (d, J = 8.4 Hz, 2H), 3.86 (q, J = 6.4 Hz, 2H), 2.87 (t, J = 6.4 Hz, 2H), 1.43 (t, J = 5.2 Hz, 1H), 1.33 (s, 12H) ppm; ^{13}C NMR (125 MHz, $CDCl_3$, 25 °C): δ = 139.28, 134.78, 132.03, 130.93, 129.28, 127.02, 126.21,

125.94, 121.47, 90.82, 89.58, 84.16, 60.60, 39.31 ppm; HR ESI-TOF MS (MeOH, positive mode): *m*/*z* = 349.1974 (calculated *m*/*z* on the basis of the monoisotropic mass of $C_{22}H_{26}BO_3$ $[M + H]^+$ = 349.1975).

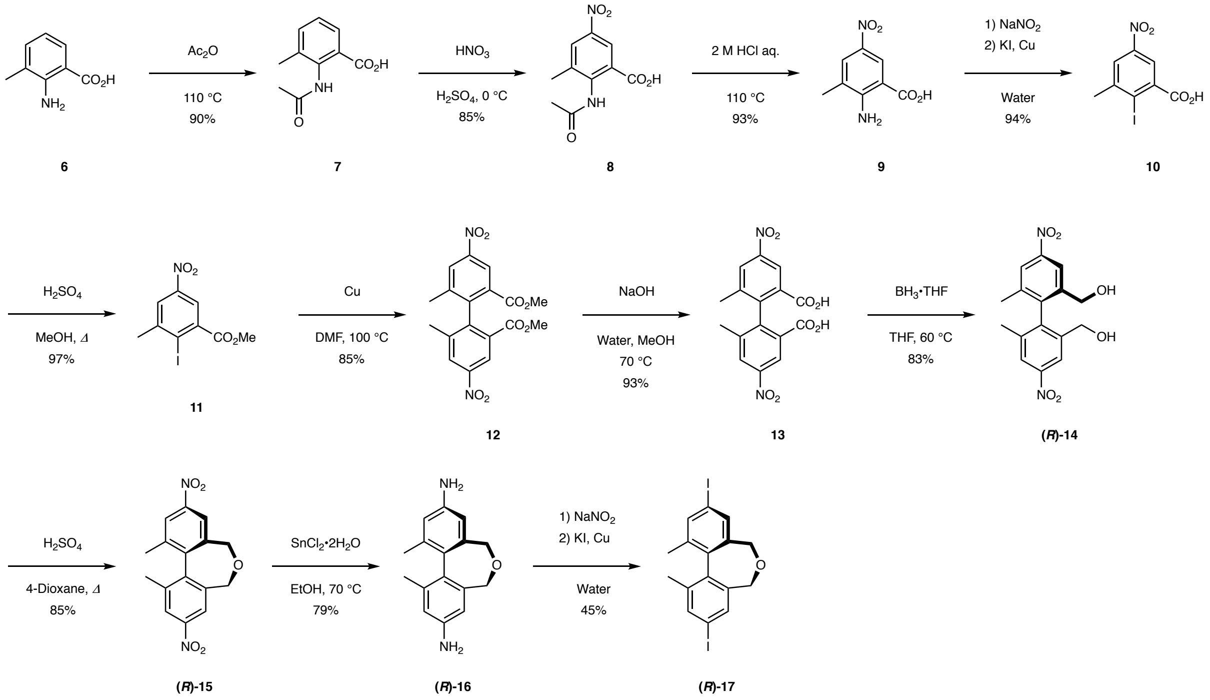

Synthesis of (*R*)-16. Compound (*R*)-**16** was synthesized from **6** by following the reported procedures.[1,2]

Optical resolution of 13. To racemic **13** (3.15 g, 8.74 mmol) was added quinine (2.84 g, 8.74 mmol) and acetone (40 mL) at 25 °C, and the mixture was heated to reflux. After being cooled to 20 °C, colorless crystals **13**•quinine precipitated out, which were collected by filtration and dried under reduced pressure. The crystals were dissolved in a mixture of Et_2O (30 mL) and 4M HCl aq. (30 mL), and the organic extract was dried over Na_2SO_4 followed by filtration from the insoluble substances through a filter paper. The filtrate was evaporated to dryness under reduced pressure, and the residual yellow solid was reprecipitated with acetone and hexane to isolate (*S*)-**13** (0.779 g, 2.16 mmol) as yellow crystals.

The colorless crystals of **13**•quinine were recrystallized in $CHCl_3$ and hexane to prepare single crystals for X-ray crystallographic analysis, which allowed for determination of the absolute configuration of the enantiomer of **13** to be *S* (Supplementary Fig. 1).

Recrystallization of the racemic **13** by following the similar procedure using quinidine in place of quinine allowed for the isolation of (*R*)-**13** in the enantiomeric excess of >99% evaluated by chromatography.

Spectroscopic data of (*R*)-13. UV-vis (MeOH, 20 °C): λ_{max} (ε) = 221 (33100), 277 (16600) nm; CD (MeOH, 20 °C): λ_{max} ($\Delta\varepsilon$) = 217 (–12.44), 236 (3.99), 289 (0.90) nm.

Spectroscopic data of (*R*)-14. UV-vis (MeOH, 20 °C): λ_{max} (ε) = 209 (28800), 279 (18100) nm; CD (MeOH, 20 °C): λ_{max} ($\Delta\varepsilon$) = 212 (–0.83), 283 (0.29) nm.

Spectroscopic data of (*R*)-15. UV-vis (MeOH, 20 °C): λ_{max} (ε) = 214 (13100), 293 (8000) nm; CD (MeOH, 20 °C): λ_{max} ($\Delta\varepsilon$) = 214 (–46.49), 230 (13.97), 244 (–2.15), 297 (7.95) nm.

Spectroscopic data of (*R*)-16. UV-vis (MeOH, 20 °C): λ_{max} (ε) = 214 (31200), 272 (18500) nm; CD (MeOH, 20 °C): λ_{max} ($\Delta\varepsilon$) = 213 (–55.30), 238 (10.09), 270 (26.15), 319 (–0.98) nm.

Synthesis of (*R*)-17. To a mixture of (*R*)-**16** (3.57 g, 14.0 mmol) and $NaNO_2$ (2.37 g, 84.3 mmol) were added 2M NaOH aq. (40 mL) at 0 °C under Ar, followed by addition of water (40 mL), 4M HCl aq. (48 mL) and 12M HCl aq. (96 mL) in this order at 0 °C. Then the resulting mixture was stirred for 1.5 h. To the mixture, was added urea (2.0 g, 33.3 mmol) to consume the residual $NaNO_2$, followed by an aqueous solution of KI (34.98 g, 210.7 mmol) and Cu (410 mg, 6.45 mmol). After being stirred for 15 h at 20 °C, the reaction mixture was filtered to remove insoluble substances. The filtrate was extracted with $CHCl_3$, and organic phase was dried over Na_2SO_4 and filtered. The filtrate was evaporated to dryness under reduced pressure at 20 °C, and the residue was chromatographed on silica gel (Silica Gel 60) with $CHCl_3$ and hexane (1/5 v/v) as an eluent, followed by reprecipitation with $CHCl_3$ and hexane to allow isolation of (*R*)-**17** (2.98 g, 6.26 mmol) as yellow crystals in 45% yield.
TLC (Merck 60 F254, CH_2Cl_2) R_f = 0.90; ^{1}H NMR (400 MHz, acetone-d_6, 22 °C): δ = 7.78 (d, J = 1.6 Hz, 2H), 7.66 (d, J = 1.6 Hz, 2H), 4.38 (d, J = 11.6 Hz, 2H), 3.85 (d, J = 11.6 Hz, 2H), 2.18 (s, 6H) ppm; ^{13}C NMR (125 MHz, acetone-d_6, 22 °C): δ = 139.12, 138.42, 137.37, 135.29, 93.45, 65.95, 18.01 ppm; HR ESI-TOF MS (MeOH, positive mode): m/z = 476.9209 (calculated m/z on the basis of the monoisotropic mass of $C_{16}H_{15}I_2O$ $[M + H]^+$ = 476.9212); UV-vis (MeOH, 20 °C): λ_{max} (ε) = 212 (35300), 260 (26600) nm; CD (MeOH, 20 °C): λ_{max} ($\Delta\varepsilon$) = 214 (–63.20), 233 (32.05), 259 (36.11) nm.

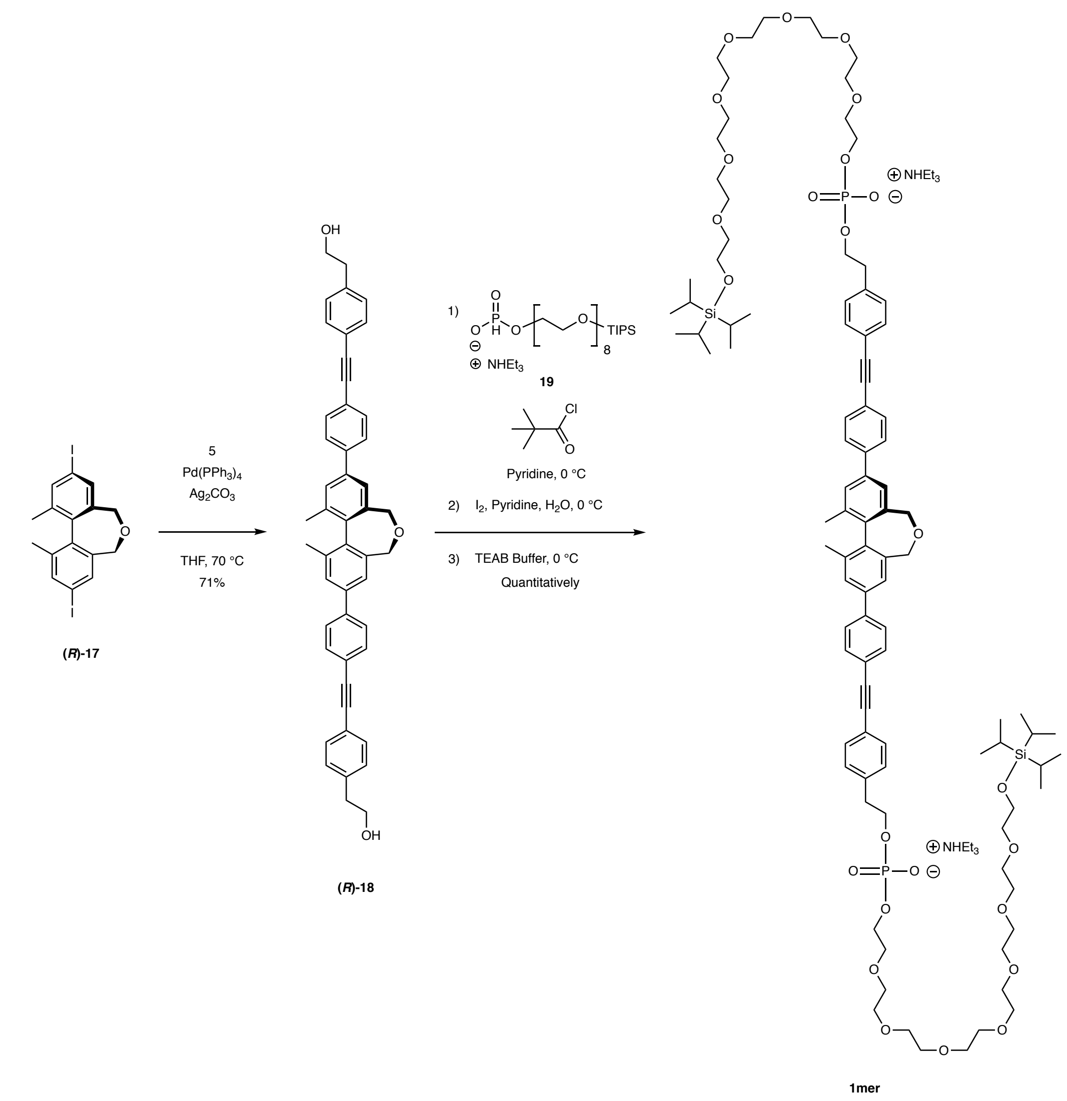

Synthesis of (*R*)-18. To a degassed dry THF (100 mL) solution of **5** (2.34 g, 6.73 mmol) was added (*R*)-**17** (1.29 g, 3.18 mmol), $Pd(PPh_3)_4$ (722 mg, 625 μmol) and Ag_2CO_3 (3.10 g, 11.2 mmol) at 20 °C under Ar, and the reaction mixture was refluxed for 26 h. Then, the mixture was evaporated to dryness under reduced pressure at 20 °C, and the residue was chromatographed on silica gel (Silica Gel 60) with $CHCl_3$ and MeOH (20/1 v/v) as an eluent, followed by reprecipitation with $CHCl_3$ and hexane to allow isolation of (*R*)-**18** (1.50 g, 2.25 mmol) as yellow crystals in 71% yield.

TLC (Merck 60 F254, $CHCl_3$/MeOH 5/1) R_f = 0.52; ^{1}H NMR (400 MHz, $CDCl_3$, 22 °C): δ = 7.66 (dd, J = 6.4 and 2.0 Hz, 4H), 7.61 (dd, J = 6.4 and 2.0 Hz, 4H), 7.58 (d, J = 1.2 Hz, 2H), 7.50 (d, J = 1.2 Hz, 2H), 7.49 (dd, J = 6.4 and 2.0 Hz, 4H), 7.22 (dd, J = 6.4 and 2.0 Hz, 4H), 4.51 (d, J = 11.6 Hz, 2H), 4.14 (d, J = 11.6 Hz, 2H), 3.88 (t, J = 6.4 Hz, 2H), 2.89 (t, J = 6.4 Hz, 2H), 2.32 (s, 6H), 1.37 (brs, 1H) ppm; HR ESI-TOF MS (MeOH, positive mode): m/z = 665.3060 (calculated m/z on the basis of the monoisotropic mass of $C_{48}H_{41}O_3$ $[M + H]^+$ = 665.3055); UV-vis (THF, 20 °C): λ_{max} (ε) = 329 (276000) nm; CD (THF, 20 °C): λ_{max} ($\Delta\varepsilon$) =

218 (–203.4), 236 (105.8), 265 (–15.4), 318 (37.7) nm.

Synthesis of 19. To a dry THF (10 mL) solution of 3,3-diisopropyl-2-methyl-4,7,10,13,16,19,22,25-octaoxa-3-silaheptacosan-27-ol (420 mg, 797 μmol) was added 4-dimethylaminopyridine (347 mg, 2.84 mmol), dry NEt_3 (0.2 mL, 1.43 mmol) and salicylchlorophosphite (423 mg, 2.09 mmol) at 0 °C under Ar. The resulting mixture was stirred for 1 h at 0 °C. Then, to the reaction mixture was added TEAB buffer (2.0 mL), and the resulting mixture was evaporated to dryness under reduced pressure. The residue was chromatographed on silica gel (Chromatorex-DIOL silica) with a gradient from CH_2Cl_2 to CH_2Cl_2/MeOH (20/1) as an eluent to afford **19** (720 mg) as yellowish oil as a crude product. Due to the instability, the product was utilized for the next reaction immediately.
^{31}P NMR (162 MHz, $CDCl_3$, 22 °C): δ = 5.59 ppm.

Synthesis of (*R*)-1mer. A dry pyridine (3 mL) solution of **19** (530 mg, *ca*. 670 μmol) and (*R*)-**18** (118 mg, 177 μmol) was evaporated under reduced pressure. To the residue was added dry pyridine (5 mL) and pivaloyl chloride (0.2 mL, 1.63 mmol) at 0 °C under Ar. The resulting mixture was stirred for 10 min at 0 °C and for 20 min at 25 °C. Then, to the reaction mixture was added a dry pyridine (2 mL) solution of I_2 (59.2 mg, 466 μmol), and the resulting mixture was stirred for 20 min at 25 °C. To the reaction mixture was added saturated $Na_2S_2O_3$ aq. until the solution color turned from brown to yellow, followed by triethylammonium bicarbonate buffer (3 mL). The resulting mixture was evaporated to dryness under reduced pressure, and the residue was chromatographed on bio-beads (S-X3) with $CHCl_3$ as an eluent to allow isolation of **(*R*)-1mer** (350 mg, 177 μmol) as yellowish oil quantitatively.
TLC (Diol TLC, $CHCl_3$/MeOH 10/1) R_f = 0.17; ^{1}H NMR (400 MHz, $CDCl_3$, 22 °C): δ = 7.62–7.56 (m, 10H), 7.46–7.41 (m, 6H), 7.24–7.18 (m, 4H), 4.48 (d, J = 10.4 Hz, 4H), 4.11 (d, J = 10.4 Hz, 4H), 3.79 (t, J = 5.6 Hz, 4H), 3.70–3.52 (m, 64H), 2.94 (m, 4H), 2.29 (s, 6H), ppm; ^{31}P NMR (162 MHz, $CDCl_3$, 22 °C): δ = 0.48 ppm; HR ESI-TOF MS (MeOH, negative mode): m/z = 919.9551 (calculated m/z on the basis of the monoisotropic mass of $C_{98}H_{144}O_{25}P_2Si_2$ [M – 2H]$^{2-}$ = 919.9522 (z = 2)); UV-vis (THF, 20 °C): λ_{max} (ε) = 325 (61600) nm; CD (THF, 20 °C): λ_{max} ($\Delta\varepsilon$) = 260 (–3.6), 319 (11.7) nm.

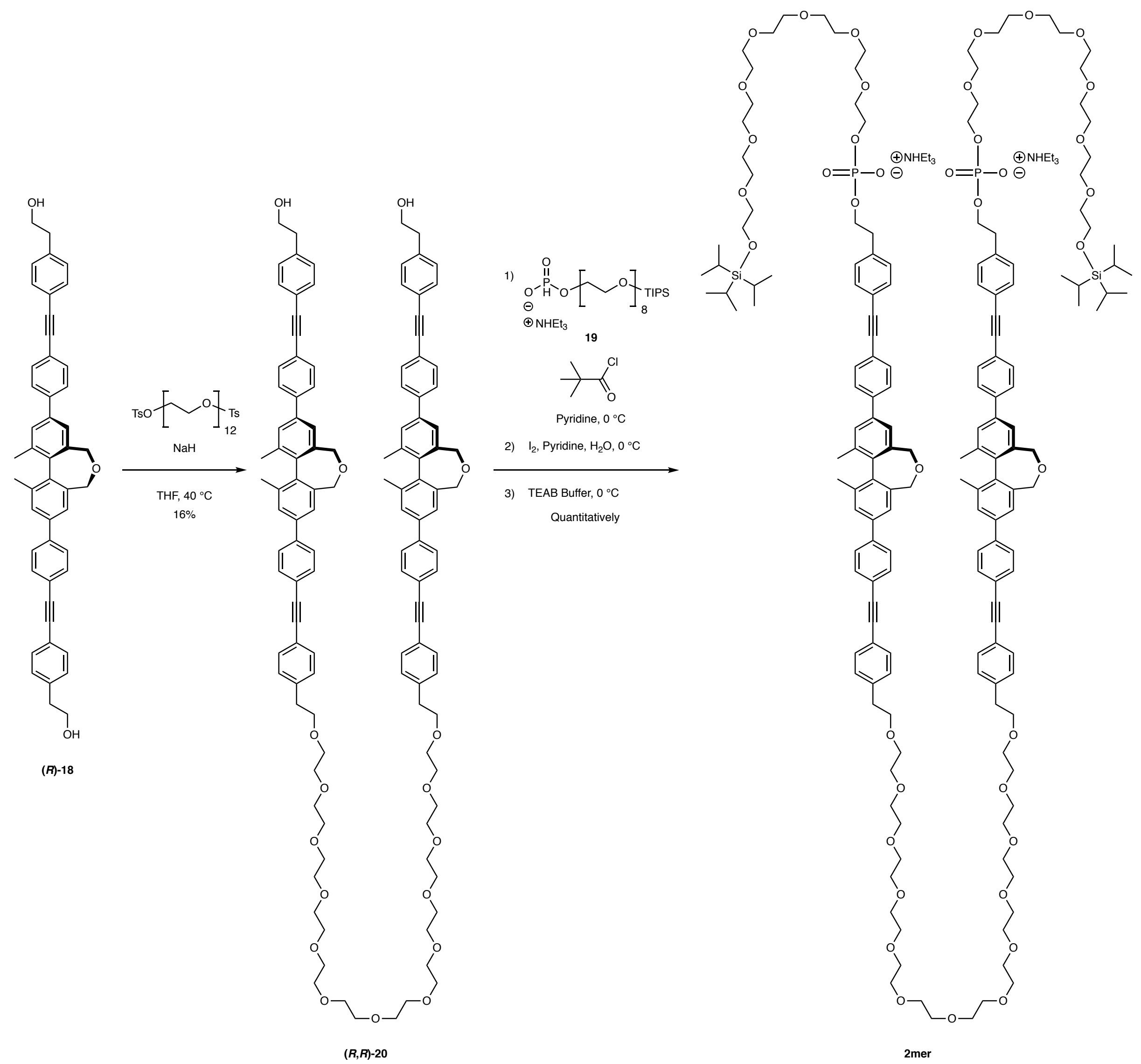

Synthesis of (*R*,*R*)-20. To a dry THF (5 mL) suspension of NaH (washed twice with dry hexanes to remove mineral oil; 68 mg, 2.83 mmol) was added a dry THF (15 mL) solution of (*R*)-**18** (624 mg, 939 μmol) and 3,6,9,12,15,18,21,24,27,30,33-undecaoxapentatriacontane-1,35-diyl bis(4-methylbenzenesulfonate) (500 mg, 584 μmol) at 0 °C under Ar, and the resulting mixture was stirred for 4.5 days at 40 °C. Then, the reaction mixture was cooled to 20 °C followed by addition of saturated NH_4Cl aq. (10 mL), and the resulting mixture was extracted with CH_2Cl_2 (20 mL, three times). The collected organic extract was dried over anhydrous Na_2SO_4 and filtered off from insoluble substances. The filtrate was evaporated to dryness under reduced pressure at 30 °C, and the residual yellow oil was chromatographed with a Japan Analytical Industry LC-9201 Recycling Preparative HPLC system with JAIGEL-1H and 2H columns for size exclusion chromatography with $CHCl_3$ as an eluent running at 3.5 mL min^{-1} to allow for isolation of (*R*,*R*)-**20** (140 mg, 75.8 μmol) in 16% yield.

^{1}H NMR (400 MHz, $CDCl_3$, 22 °C): δ = 7.65 (dd, J = 8.8 and 2.0, 8H), 7.60 (dd, J = 8.8 and

2.0, 8H), 7.58 (d, J = 1.6 Hz, 4H), 7.49 (dd, J = 6.4 and 1.6 Hz, 8H), 7.45 (d, J = 8.0 Hz, 4H), 7.23 (d, J = 1.6 Hz, 4H), 7.20 (d, J = 8.0 Hz, 4H), 4.50 (d, J = 11.2 Hz, 4H), 4.13 (d, J = 11.2 Hz, 4H), 3.87 (q, J = 4.0 Hz, 4H), 3.68 (t, J = 6.4 Hz, 4H), 3.67–3.60 (m, 44H), 2.89 (q, J = 6.4 Hz, 8H), 2.31 (s, 12H) ppm; MALDI-TOF MS (CHCA, positive mode): m/z = 1862.43 (calculated m/z on the basis of the monoisotropic mass of $C_{120}H_{126}O_{17}Na$ $[M + Na]^+$ = 1862.89).

Synthesis of (*R*)-2mer. A dry pyridine (3 mL) solution of **19** (270 mg, *ca*. 220 μmol) and (*R*,*R*)-**20** (33.9 mg, 18.4 μmol) was evaporated to dryness under reduced pressure. To the residue was added dry pyridine (5 mL) and pivaloyl chloride (0.1 mL, 813 μmol) at 0 °C under Ar. The resulting mixture was stirred for 30 min at 0 °C and for 20 min at 25 °C. Then, to the reaction mixture was added a dry pyridine (2 mL) solution of I_2 (59.2 mg, 466 μmol), and the resulting mixture was stirred for 20 min at 25 °C. To the reaction mixture was added saturated $Na_2S_2O_3$ aq. until the solution color turned from brown to yellow, followed by addition of triethylammonium bicarbonate buffer (1 mL). The resulting mixture was evaporated to dryness under reduced pressure, and the residue was chromatographed on bio-beads (S-X1) with $CHCl_3$ as an eluent to allow isolation of **(*R*)-2mer** (71 mg, 23.6 μmol) as yellowish oil quantitatively.

^{1}H NMR (400 MHz, $CDCl_3$, 22 °C): δ = 7.64–7.56 (m, 20H), 7.47–7.43 (m, 12H), 7.24–7.18 (m, 8H), 4.48 (d, J = 10.4 Hz, 4H), 4.11 (d, J = 10.4 Hz, 4H), 3.79 (t, J = 5.2 Hz, 4H), 3.70–3.50 (m, 120H), 2.87 (t, J = 6.4 Hz, 8H), 2.29 (s, 12H) ppm; ^{13}C NMR (125 MHz, $CDCl_3$, 22 °C): δ = 132.22, 131.75, 129.17, 127.04, 125.40, 72.87, 72.07, 70.72, 70.58, 70.49, 67.84, 20.17, 18.12, 12.10 ppm; ^{31}P NMR (162 MHz, $CDCl_3$, 22 °C): δ = 0.48 ppm; HR ESI-TOF MS (MeOH, negative mode): m/z = 1507.758 (calculated m/z on the basis of the monoisotropic mass of $C_{170}H_{230}O_{39}P_2Si_2$ $[M – 2H]^{2–}$ = 1507.754 (z = 2)); UV-vis (THF, 20 °C): λ_{max} (ε) = 329 (88300) nm; CD (THF, 20 °C): λ_{max} ($\Delta\varepsilon$) = 264 (–7.3), 319 (14.5) nm.

Supplementary Figures (Supplementary Figs. 1–37)

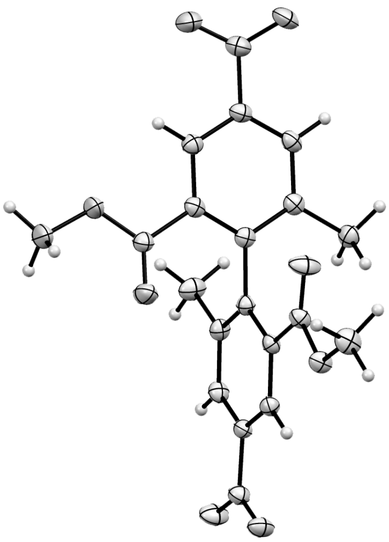

Supplementary Fig. 1 | X-ray crystallographic analysis of 12 (racemic).

ORTEP drawing of **12** at 110 K (50 % thermal ellipsoids). Crystal data (deposition number CCDC 1887565): $C_{18}H_{16}N_2O_8$, M_w = 388.33, monoclinic, $P2_1/c$, a = 13.1456(2), b = 8.3321(2), c = 16.3360(3) Å, β = 90.3100(10)°, V = 1789.26(6) Å^3, Z = 4, ρ_{calcd} = 1.442 g cm^{-3}, μ = 9.85 cm^{-1}, F000 = 808, theta range 3.362 to 68.182°, Cu$_{K\alpha}$ λ = 1.54187 Å, T = 110(2) K, No. of unique reflections = 3263 (R_{int} = 0.0248), GOF = 1.052, $R1$ = 0.0391 ($I > 2\sigma(I)$), $wR2$ = 0.1023 ($I > 2\sigma(I)$).

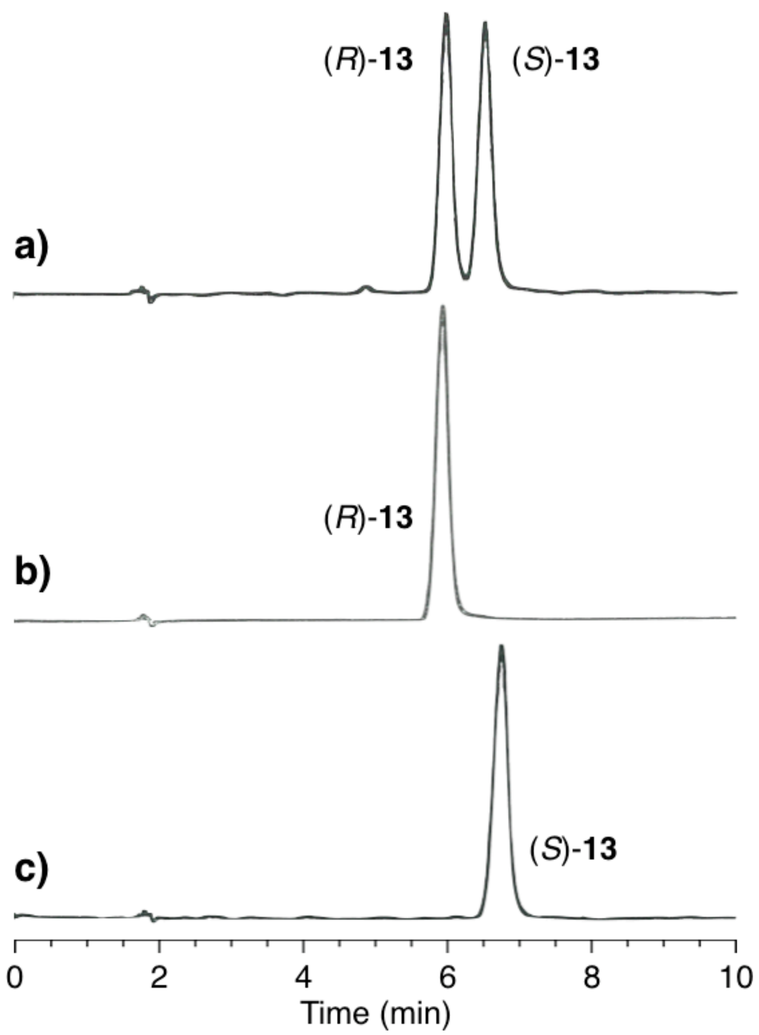

Supplementary Fig. 2 | Optical resolution of 13.

HPLC traces of a) racemic **13**, b) (*R*)-**13** obtained by crystallization with quinidine and c) (*S*)-**13** obtained by crystallization with quinine using CHIRALPAK IC column (Daicel, inner diameter: 4.6 mm, length: 250 mm). Eluent: hexane/EtOH /trifluoroacetic acid = 95/5/0.1 (v/v/v). Flow rate: 2.0 mL min^{-1}. Detection: absorption at 275 nm. Temperature: 25 °C. Samples were dissolved in EtOH in the concentration of 1.0 mg mL^{-1}, and 5 μL solution was injected.

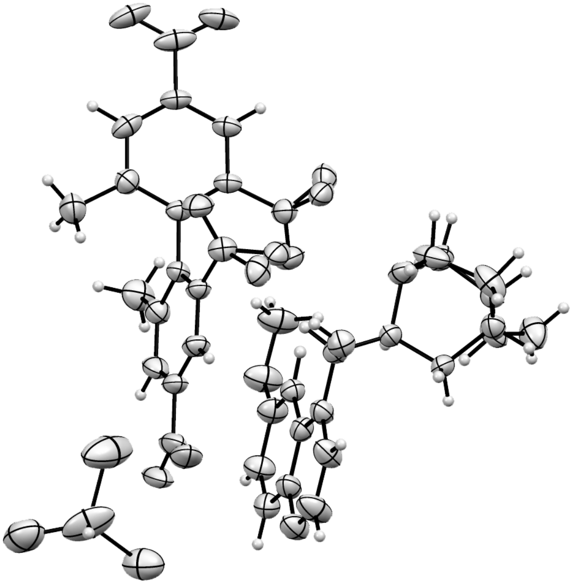

Supplementary Fig. 3 | X-ray crystallographic analysis of (*S*)-13•quinine•$CHCl_3$.

ORTEP drawing of (*S*)-**13**• quinine•$CHCl_3$ at 110 K (50 % thermal ellipsoids). Crystal data (deposition number CCDC 1043282): $C_{58}H_{62}Cl_6N_6O_{12}$, M_w = 1247.83, orthorhombic, *C2221*, a = 13.5315(3), b = 16.9652(3), c = 25.6887(5) Å, $\alpha = \beta = \gamma = 90°$, V = 5897.2(2) Å^3. Z = 4, ρ_{calcd} = 1.405 g cm^{-3}, μ = 3.214 cm^{-1}, F000 = 2600, theta range 3.441 to 68.235°, $Cu_{K\alpha}$ λ = 1.54187 Å, T = 110(2) K, No. of unique reflections = 5368 (R_{int} = 0.0599), GOF = 1.075, $R1$ = 0.0816 ($I > 2\sigma(I)$), $wR2$ = 0.2294 ($I > 2\sigma(I)$).

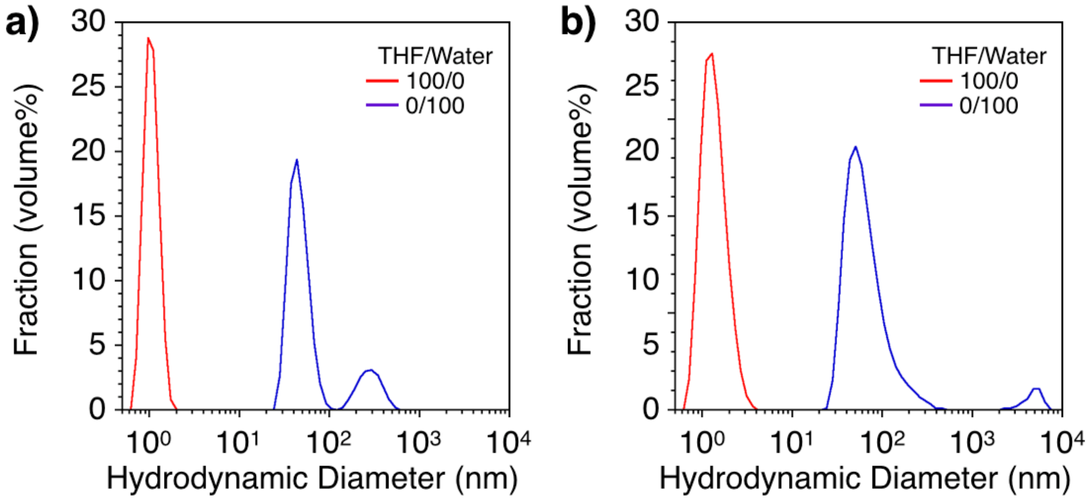

Supplementary Fig. 4 | DLS analysis of 1mer and 2mer in THF and water.

DLS profiles of a) **1mer** and b) **2mer** in THF (red) and water (blue) at 20 °C. [**1mer**] = 14 μM. [**2mer**] = 7.0 μM.

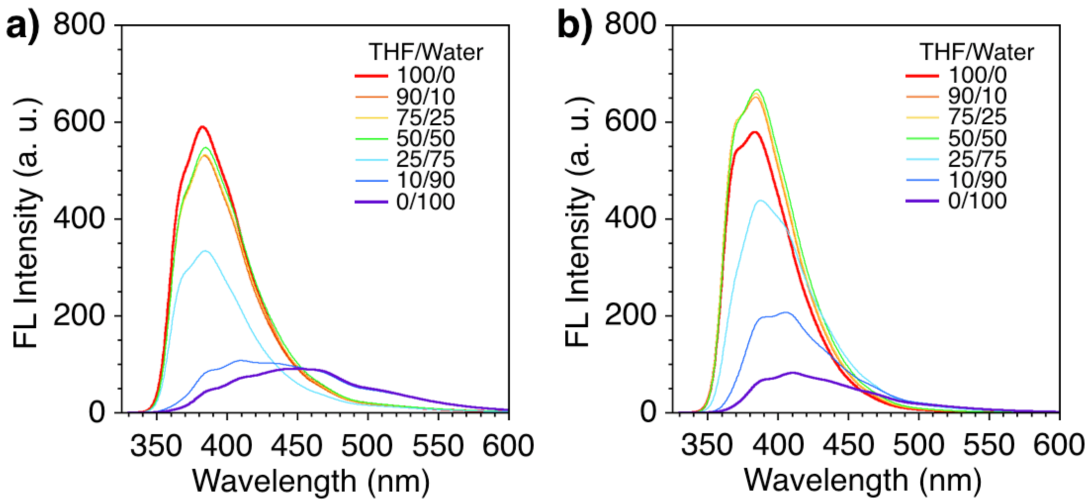

Supplementary Fig. 5 | Fluorescence study of 1mer and 2mer in THF and water.

Fluorescence spectra of a) **1mer** and b) **2mer** in the mixture of THF and water at 20 °C. [**1mer**] = 14 μM. [**2mer**] = 7.0 μM. Excitation at λ = 325 nm.

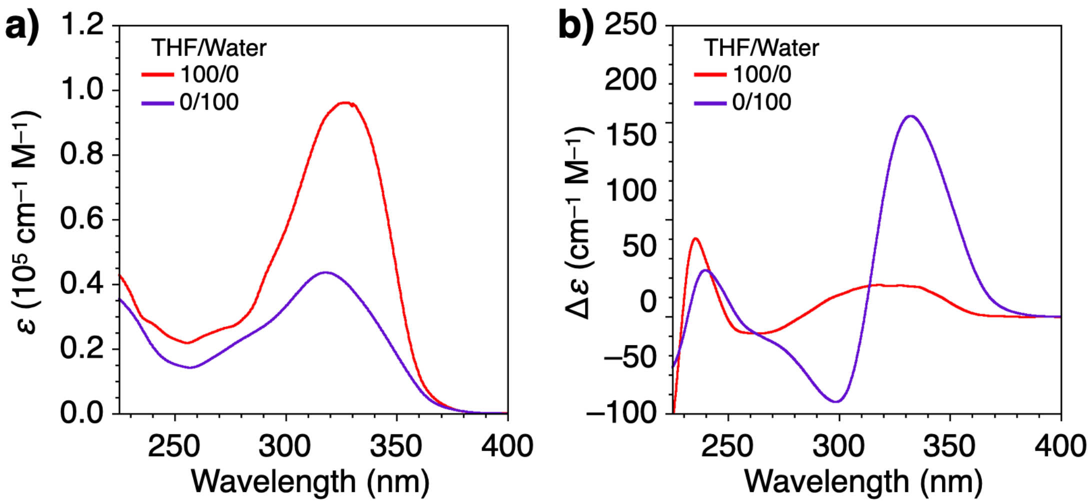

Supplementary Fig. 6 | UV-vis absorption and CD spectral studies of 2mer in THF and water.

a) UV-vis absorption and b) CD spectra of **2mer** in THF (red) and water (blue) at 20 °C. [**2mer**] = 2.5 mM.

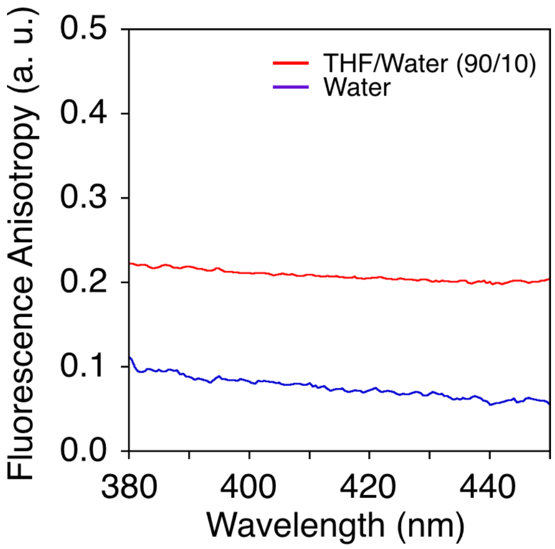

Supplementary Fig. 7 | Fluorescence anisotropy of 2mer.

Depolarization measurements of emission of **2mer** in the mixture of THF and water (red) and in water (blue) at 20 °C. [**2mer**] = 7.0 μM. Excitation at λ = 325 nm.

Fluorescence anisotropy r of **2mer** decreased upon increment of the solvent polarity (r = 0.218 in THF/water = 90/10, and 0.087 in water at 390 nm). In general, a slowly tumbling object, in other words a larger object, shows a larger fluorescence anisotropy value.[3] Since the solvent polarity increment elicited the aggregation formation, the observed decrease in the fluorescence anisotropy indicates energy transfer, namely, excimer formation of the BPO units of **2mer**.

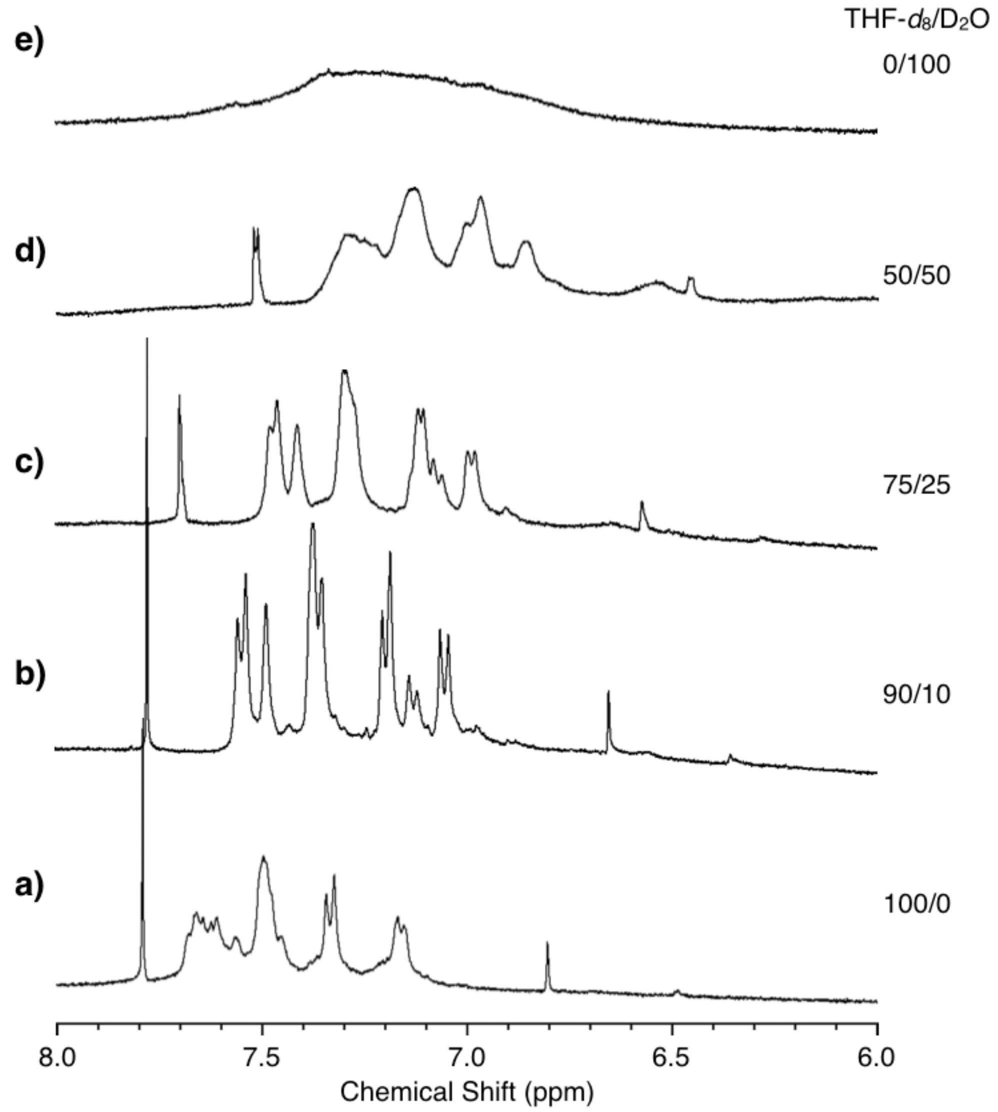

Supplementary Fig. 8 | ^{1}H NMR spectral study of 2mer.

^{1}H NMR spectra of **2mer** in a mixture of THF-d_8 and D_2O at a) 100/0, b) 90/10, c) 75/25, d) 50/50 and e) 0/100 at 20 °C. [**2mer**] = 2.5 mM.

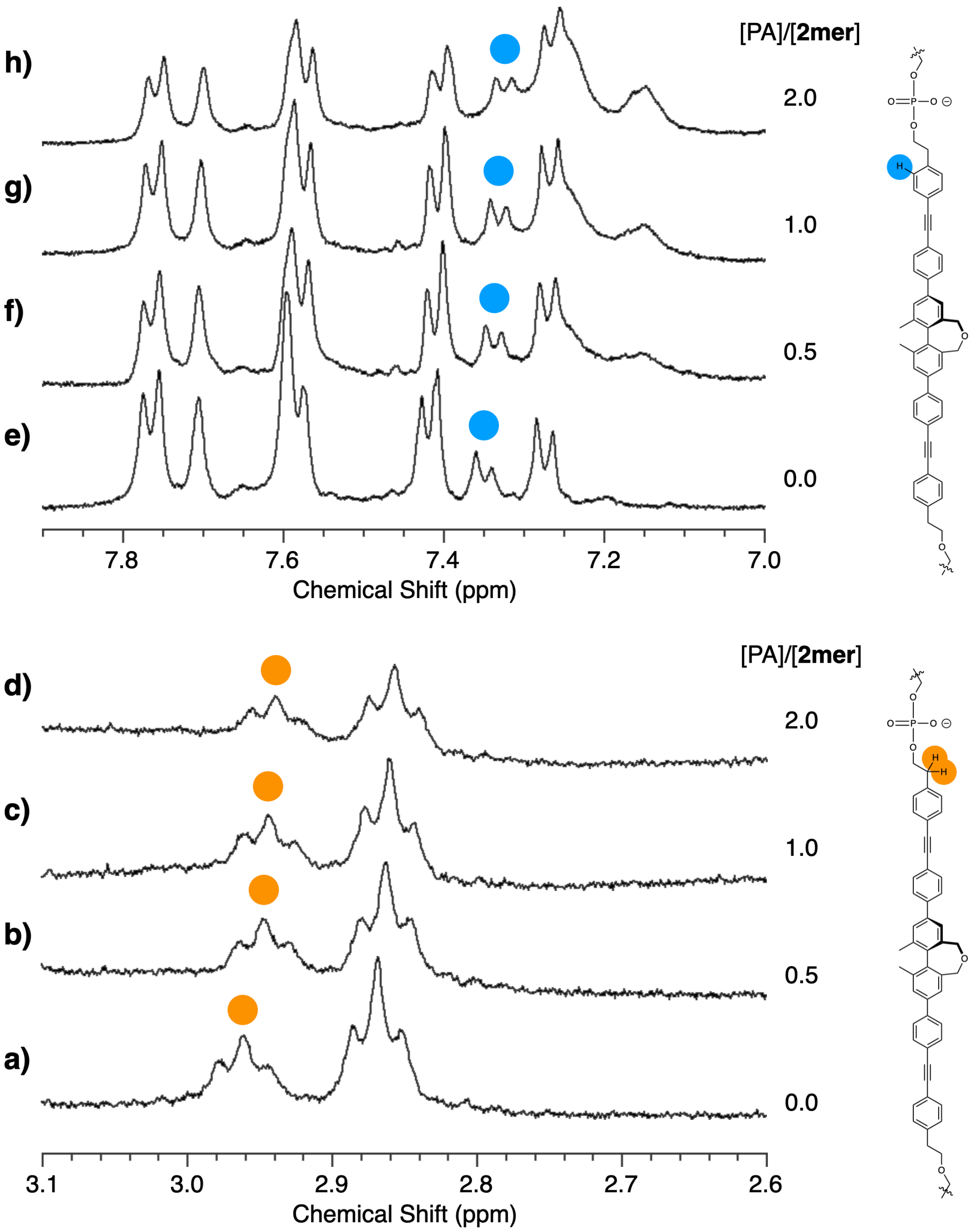

Supplementary Fig. 9 | ^{1}H NMR spectral study of 2mer upon addition of PA.

^{1}H NMR spectra of **2mer** in THF-d_8/D_2O (90/10) at the ranges of a–d) 3.1–2.6 and e–f) 7.9–7.0 ppm upon addition of PA at [PA]/[**2mer**] = a,e) 0.0, b,f) 0.5, c,g) 1.0 and d,h) 2.0 at 24 °C. The blue and orange marks in the spectra denote the signals corresponding to the protons highlighted by the same marks in the chemical formulae.

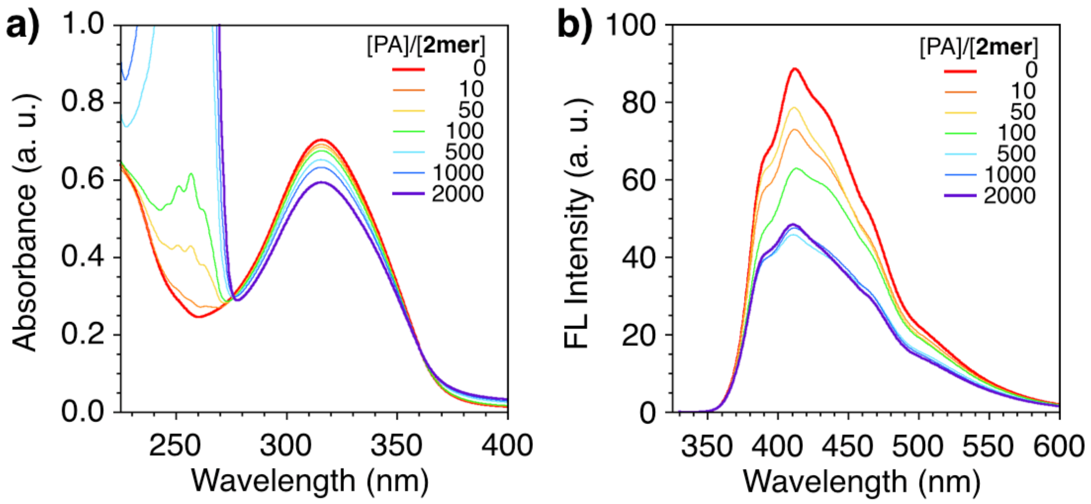

Supplementary Fig. 10 | UV-vis absorption and fluorescence spectral studies of 2mer upon addition of PA.

a) UV-vis absorption and b) fluorescence spectral changes of **2mer** ([**2mer**] = 10 μM) in HEPES buffer at 20 °C upon titration with PA at [PA]/[**2mer**] = 0.0, 10, 50, 100, 500, 1000 and 2000. Excitation at λ = 325 nm. 20 mM HEPES, 50 mM KCl, 2.0 mM $MgCl_2$, pH 7.5.

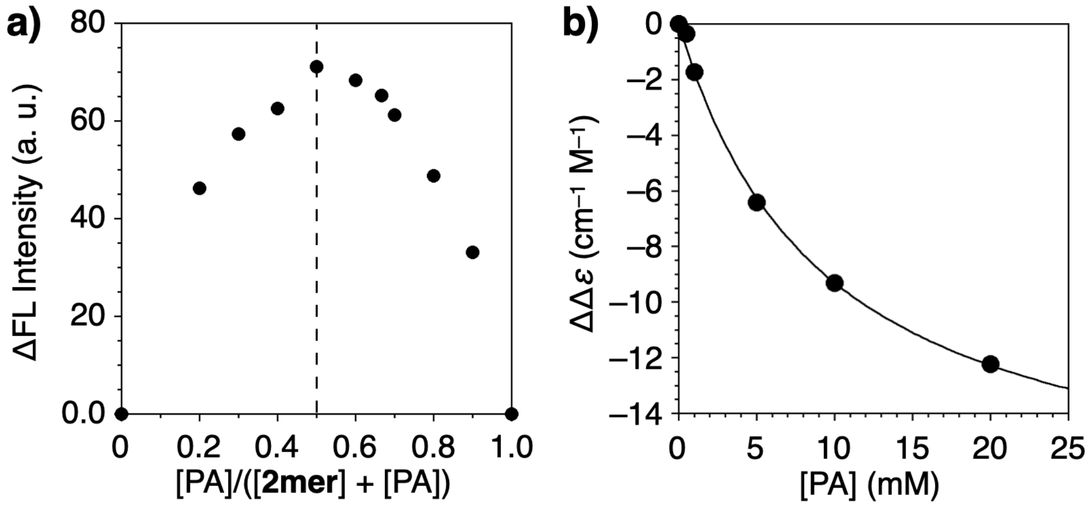

Supplementary Fig. 11 | Job plot and curve fitting analysis of titration between 2mer and PA.

a) Job plot for evaluating stoichiometry of the complexation between **2mer** and PA in HEPES buffer on the basis of the fluorescence signal intensity (390.0 nm). b) Curve fitting analysis of the CD signal change (320.0 nm) of **2mer** upon titration with PA in HEPES buffer. R = 0.999.

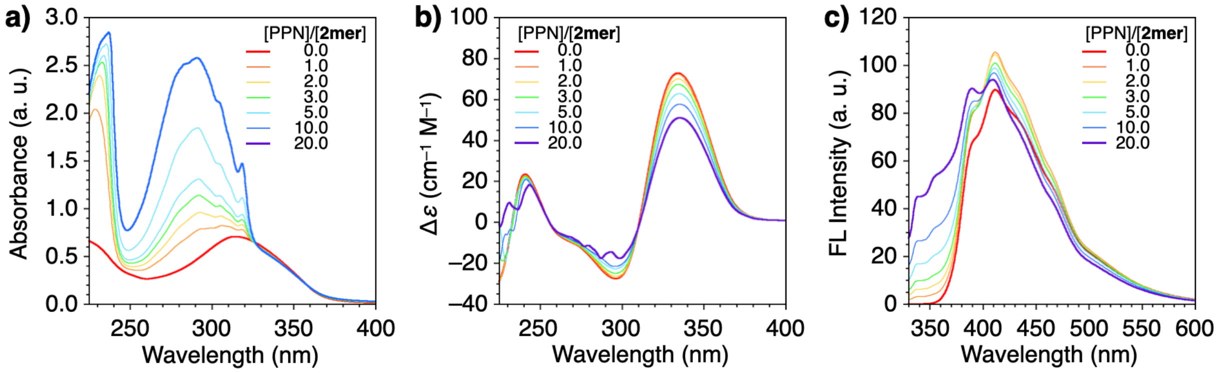

Supplementary Fig. 12 | UV-vis absorption, CD and fluorescence spectral studies of 2mer upon addition of PPN.

a) UV-vis absorption, b) CD and c) fluorescence spectral changes of **2mer** ([**2mer**] = 10 μM) in HEPES buffer at 20 °C upon titration with PPN at [PPN]/[**2mer**] = 0.0, 1.0, 2.0, 3.0, 5.0, 10.0 and 20.0. Excitation at λ = 325 nm. 20 mM HEPES, 50 mM KCl, 2.0 mM $MgCl_2$, pH 7.5.

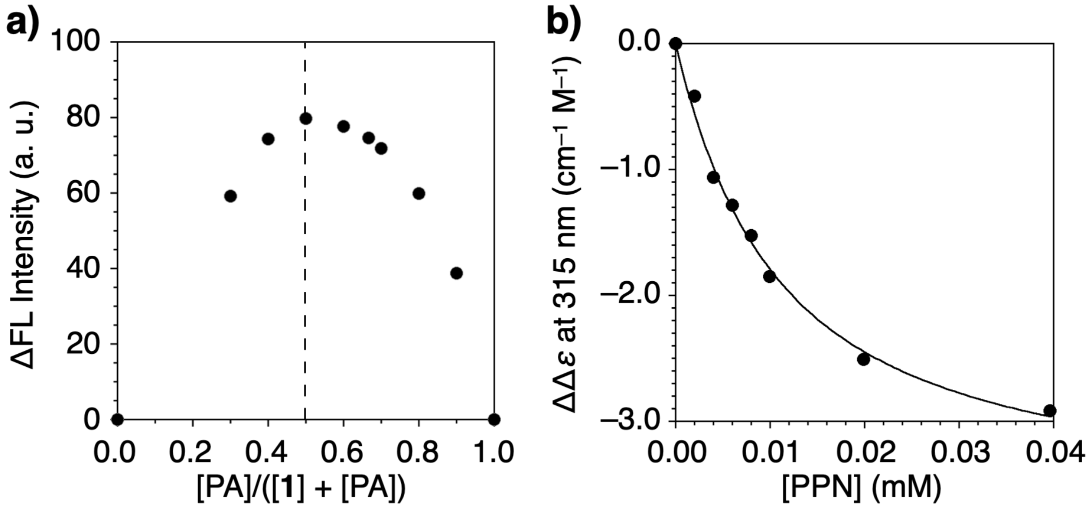

Supplementary Fig. 13 | Job plot and curve fitting analysis of complexation between membrane- embedded 2mer and PPN.

a) Job plot for evaluating stoichiometry of the complexation of membrane-embedded **2mer** with PPN on the basis of the fluorescence signal intensity (390.0 nm). b) Curve fitting analysis of the CD signal change at 315 nm in the complexation of membrane-embedded **2mer** with PPN.

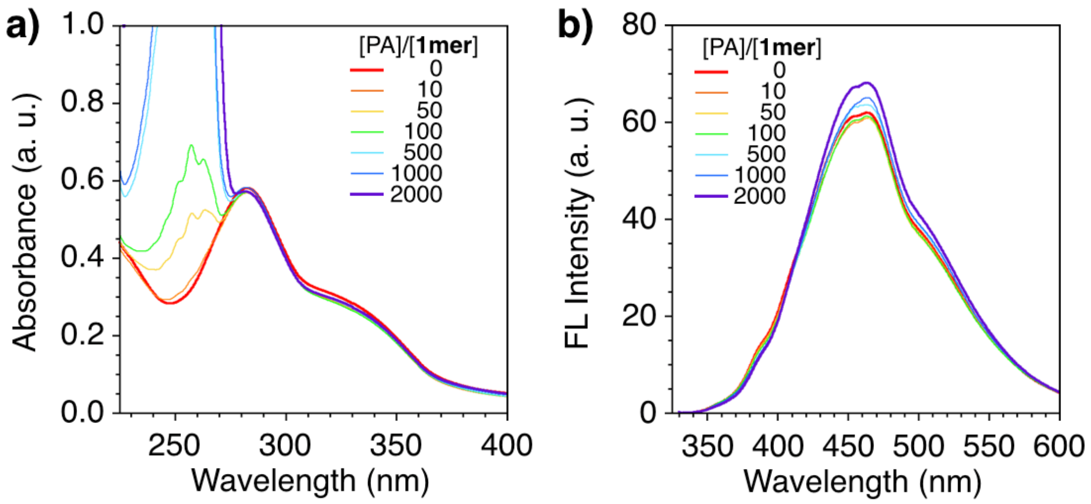

Supplementary Fig. 14 | UV-vis absorption and fluorescence spectral studies of 1mer upon addition of PA.

a) UV-vis absorption and b) fluorescence spectral changes of **1mer** ([**1mer**] = 20 μM) in HEPES buffer at 20 °C upon titration with PA at [PA]/[**1mer**] = 0.0, 10, 50, 100, 500, 1000 and 2000. Excitation at λ = 325 nm. 20 mM HEPES, 50 mM KCl, 2.0 mM $MgCl_2$, pH 7.5.

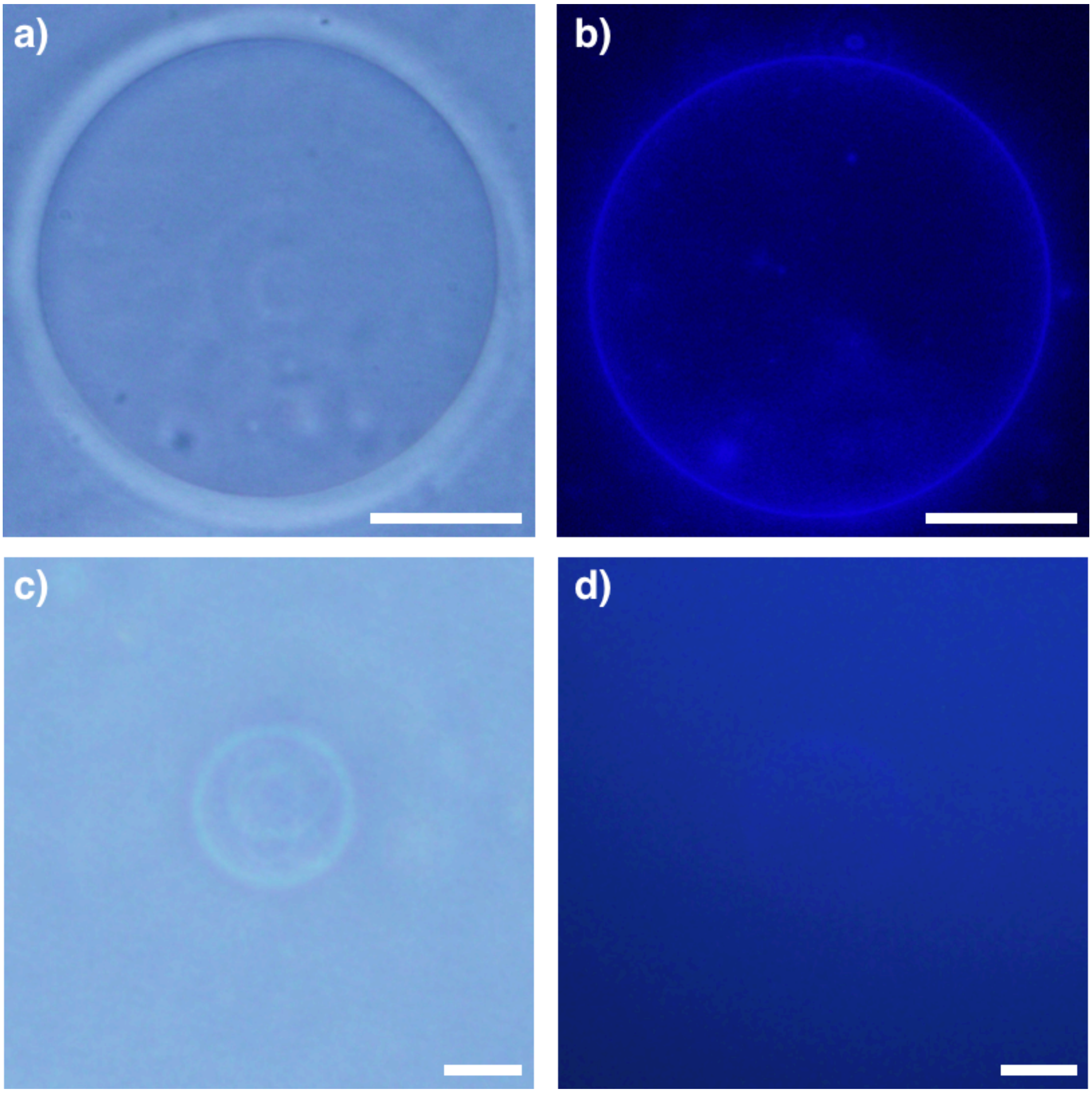

Supplementary Fig. 15 | Optical microscopic observations of DOPC•1mer GUV.
a) Phase-contrast and b) fluorescence micrographs of a DOPC•**1mer**^{pre} GUV at 20 °C that was prepared by evaporation of a $CHCl_3$ solution of a mixture of DOPC and **1mer** followed by hydration (pre-loading method, [DOPC] = 200 µM, [**1mer**] = 20 µM, intravesicular medium: 200 mM sucrose aq., extravesicular medium: a mixture of 33 mM sucrose aq. and 167 mM glucose aq.). c) Phase-contrast and d) fluorescence micrographs of a DOPC GUV after addition of **1mer** in an aqueous medium at 20 °C (post-loading method, [DOPC] = 200 µM, [**1mer**] = 20 µM, intravesicular medium: 200 mM sucrose aq., extravesicular medium: a mixture of 33 mM sucrose aq. and 167 mM glucose aq.). The fluorescence micrograph was taken by monitoring at $\lambda > 420$ nm upon excitation with 330–385 nm light. Scale bars: 10 µm.

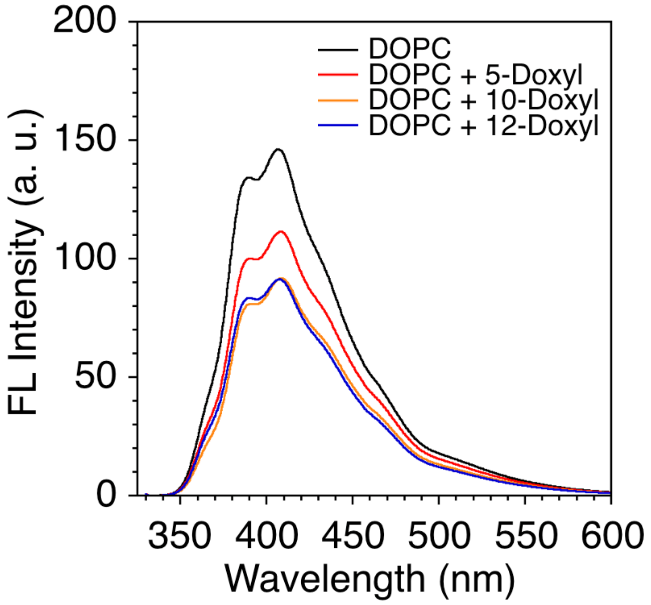

Supplementary Fig. 16 | Fluorescence depth quenching study of DOPC•1mer LUVs (pre-loadng).

Fluorescence spectra of LUVs composed of phosphocholines ([total phosphocholines] = 200 μM) containing **1mer** (20 μM) in HEPES buffer at 20 °C upon excitation at *λ* = 325 nm. The phosphocholines used for the vesicles were DOPC (black line), and mixtures of DOPC with 10-mol% 5-, 10- or 12-Doxyl PC (red, orange, and blue lines, respectively). The LUVs were prepared by evaporation of a $CHCl_3$ solution of a mixture of phosphocholines and **1mer** followed by hydration and extrusion (pre-loading method).

Incorporation of 10-mol% 5-, 10- and 12-Doxyl PCs into DOPC•**1mer** LUVs resulted in 24, 37 and 37% decreases of the fluorescence intensity of **1mer** at 407 nm, respectively.

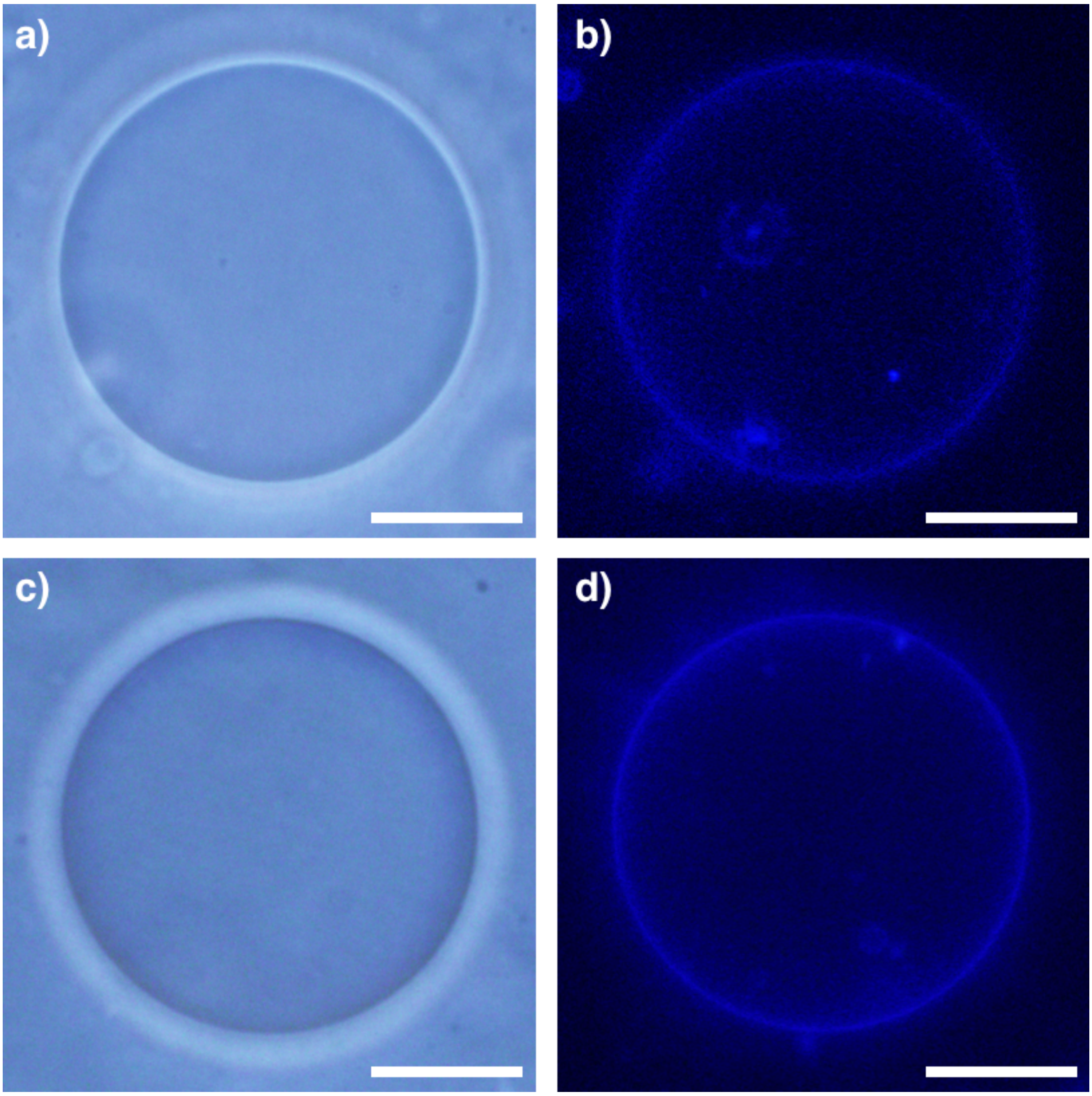

Supplementary Fig. 17 | Optical microscopic observations of DOPC•2mer GUV. a,c) Phase-contrast and b,d) fluorescence micrographs of a,b) DOPC•**2mer**^{pre} GUV and c,d) DOPC•**2mer**^{post} GUV at 20 °C ([DOPC] = 200 μM, [**2mer**] = 10 μM, intravesicular medium: 200 mM sucrose aq., extravesicular medium: a mixture of 33 mM sucrose aq. and 167 mM glucose aq.). The fluorescence micrograph was taken by monitoring at λ > 420 nm upon excitation with 330–385 nm light. Scale bars: 10 μm.

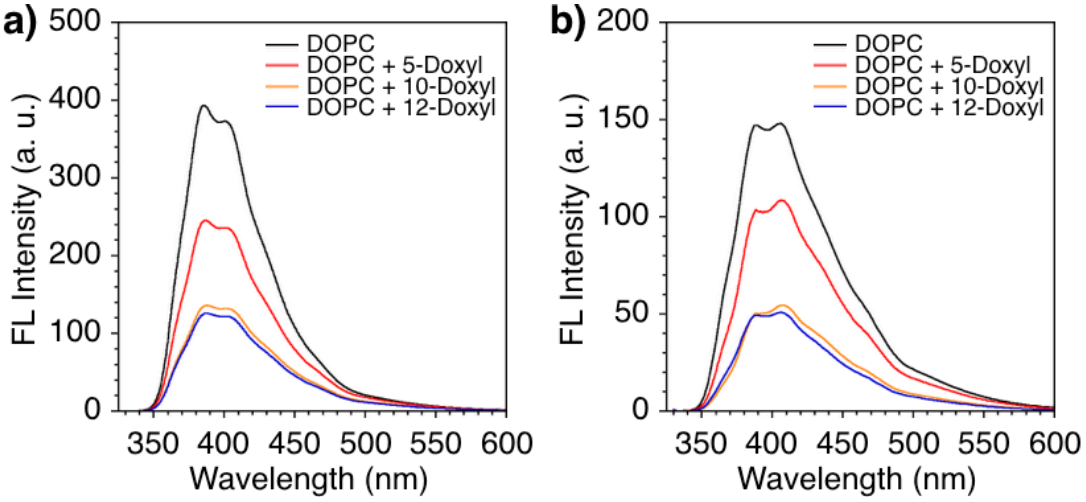

Supplementary Fig. 18 | Fluorescence depth quenching study of DOPC•2mer LUVs.

Fluorescence spectra of LUVs composed of phosphocholines ([total phosphocholines] = 200 µM) containing **2mer** (10 µM) in HEPES buffer at 20 °C upon excitation at λ = 325 nm. The phosphocholines used for the vesicles were DOPC (black line), and mixtures of DOPC with 10-mol% 5-, 10- or 12-Doxyl PC (red, orange, and blue lines, respectively). The LUVs were prepared by a) the pre-loading and b) the post-loading methods.

Incorporation of 10-mol% 5-, 10- and 12-Doxyl PCs into DOPC•**2mer** LUVs resulted in a) 37, 65 and 68% and b) 29, 64 and 66% decreases of the fluorescence intensity of **2mer** at 407 nm, respectively.

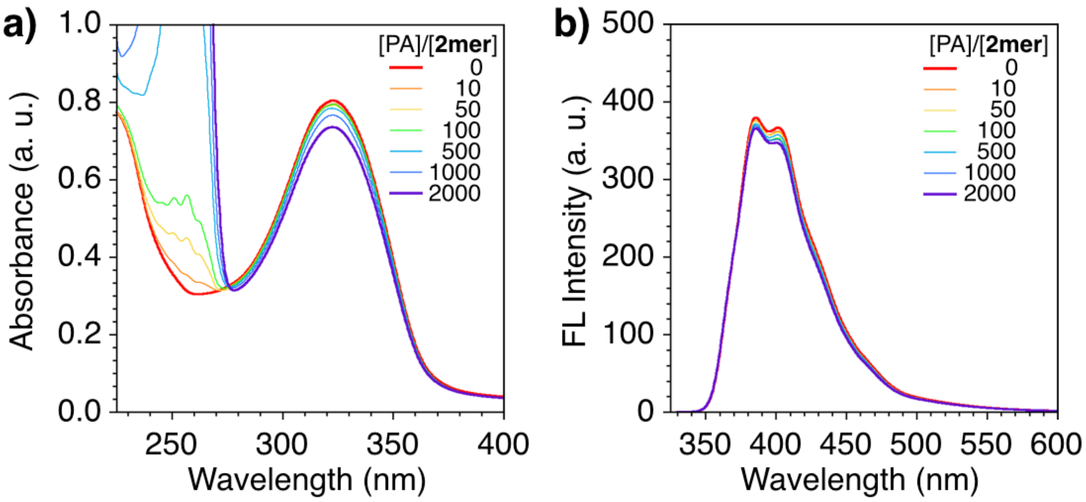

Supplementary Fig. 19 | UV-vis absorption and fluorescence spectral studies of DOPC•2mer LUVs (pre-loading) upon addition of PA.

a) UV-vis absorption and b) fluorescence spectral changes of DOPC•**2mer**^{pre} LUVs ([DOPC] = 200 μM, [**2mer**] = 10 μM) in HEPES buffer at 20 °C upon titration with PA at [PA]/[**2mer**] = 0, 10, 50, 100, 500, 1000 and 2000. PA was added to the extravesicular medium of the LUVs. Excitation at λ = 325 nm. 20 mM HEPES, 50 mM KCl, 2.0 mM $MgCl_2$, pH 7.5.

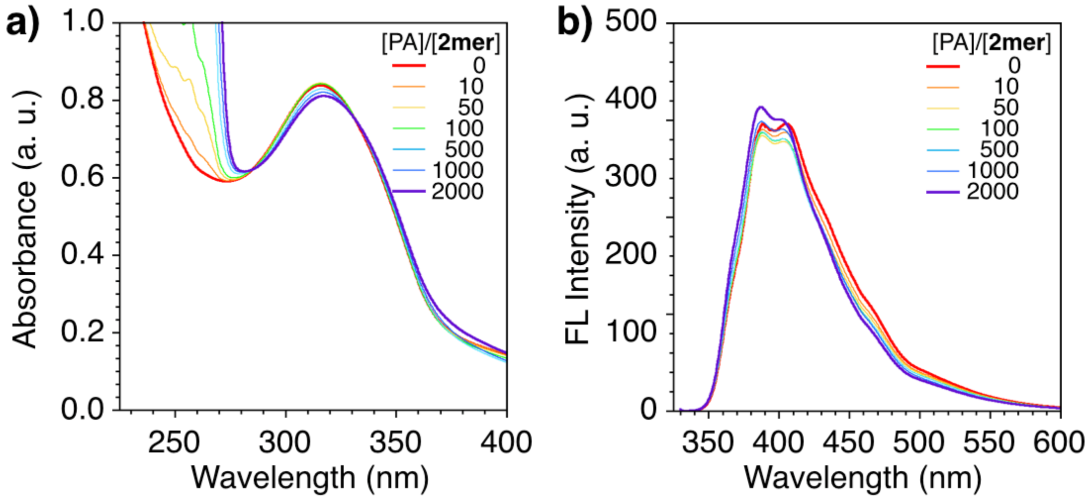

Supplementary Fig. 20 | UV-vis absorption and fluorescence spectral studies of DOPC•2mer LUVs (post-loading) upon addition of PA.

a) UV-vis absorption and b) fluorescence spectral changes of DOPC•**2mer**^{post} LUVs ([DOPC] = 200 μM, [**2mer**] = 10 μM) in HEPES buffer at 20 °C upon titration with PA at [PA]/[**2mer**] = 0, 10, 50, 100, 500, 1000 and 2000. PA was added to the extravesicular medium of the LUVs. Excitation at λ = 325 nm. 20 mM HEPES, 50 mM KCl, 2.0 mM $MgCl_2$, pH 7.5.

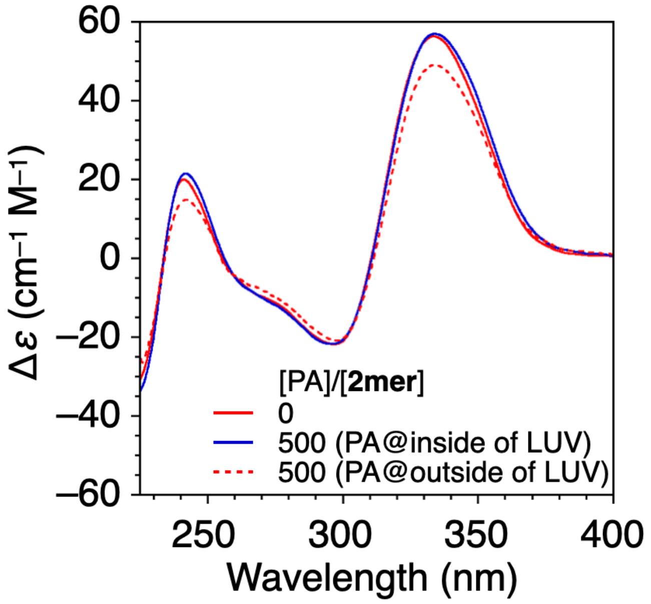

Supplementary Fig. 21 | CD spectral study of DOPC•2mer LUVs (post-loading) in the presence of PA.

CD spectra of DOPC•**2mer**^{post} LUVs with and without PA ([DOPC] = 200 μM, [**2mer**] = 10 μM) in HEPES buffer at 20 °C. Red solid line: [PA]/[**2mer**] = 0, blue solid line: [PA]/[**2mer**] = 500 (PA is inside of LUV), red broken line: [PA]/[**2mer**] = 500 (PA is outside of LUV). 20 mM HEPES, 50 mM KCl, 2.0 mM $MgCl_2$, pH 7.5.

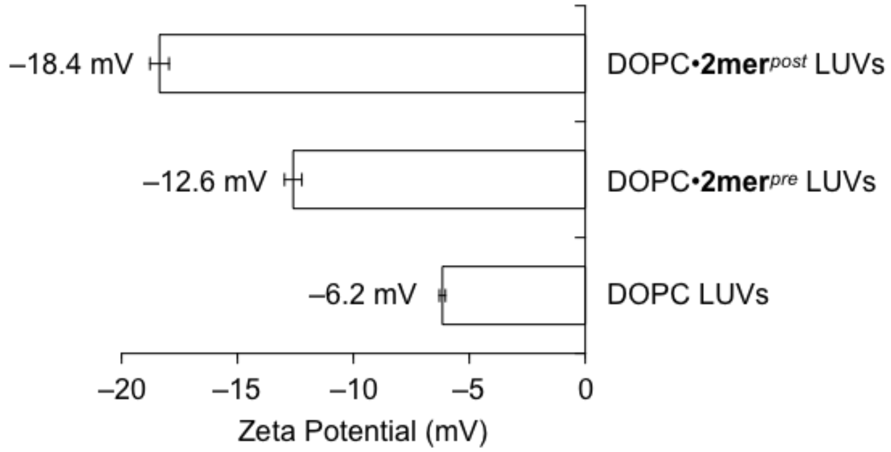

Supplementary Fig. 22 | Orientation-selectivity of 2mer in DOPC bilayer (post-loading).

Zeta potential of DOPC LUVs, DOPC•**2mer**^{pre} LUVs, and DOPC•**2mer**^{post} LUVs (200 µM DOPC, 10 µM **2mer**) in HEPES buffer (20 mM HEPES, 50 mM KCl, 2.0 mM $MgCl_2$, pH7.5) at 20 °C. Data are means of three independent experiments.

Under pH 7.5, phosphate groups are deprotonated and they are negatively charged. Thus, introduction of **2mer** into the lipid bilayers changes the surface charge of LUVs. Here we hypothesized that unidirectional introduction of **2mer** into the lipid bilayers may result in different surface charge of LUVs in comparison with that of LUVs containing non-oriented **2mer**. Because zeta potential can provide the information about surface charge of LUVs, we have prepared DOPC•**2mer** LUVs through pre-loading and post-loading methods, and compared their zeta potential. As shown in Supplementary Fig. 22, DOPC•**2mer**^{pre} LUVs, DOPC•**2mer**^{post} LUVs, and DOPC LUVs without **2mer** showed zeta potential of –12.6 ± 0.37 mV, –18.4 ± 0.41 mV, and –6.2 ± 0.13 mV, respectively. We then estimated the orientation-selectivity of **2mer** using these values. For DOPC•**2mer**^{pre} LUVs, 50% of **2mer** is supposedly oriented to the extravesicular medium and the other 50% is oriented to the intravesicular medium.[4] Therefore, when **2mer** is exclusively oriented to the extravesicular medium, its zeta potential should be the double of DOPC•**2mer**^{pre} LUVs. Based on this assumption, the orientation-selectivity *OS* (%) for DOPC•**2mer**^{post} LUVs was calculated as follows:

$$OS = \frac{\zeta_{\mathrm{post}} - \zeta_0}{2(\zeta_{\mathrm{pre}} - \zeta_0)} \times 100 \qquad (3)$$

where ζ_0, ζ_{pre}, and ζ_{post} represent zeta potential of DOPC LUVs without **2mer**, DOPC•**2mer**^{pre} LUVs, and DOPC•**2mer**^{post} LUVs, respectively. The *OS* for DOPC•**2mer**^{post} LUVs was therefore calculated to be 95%.

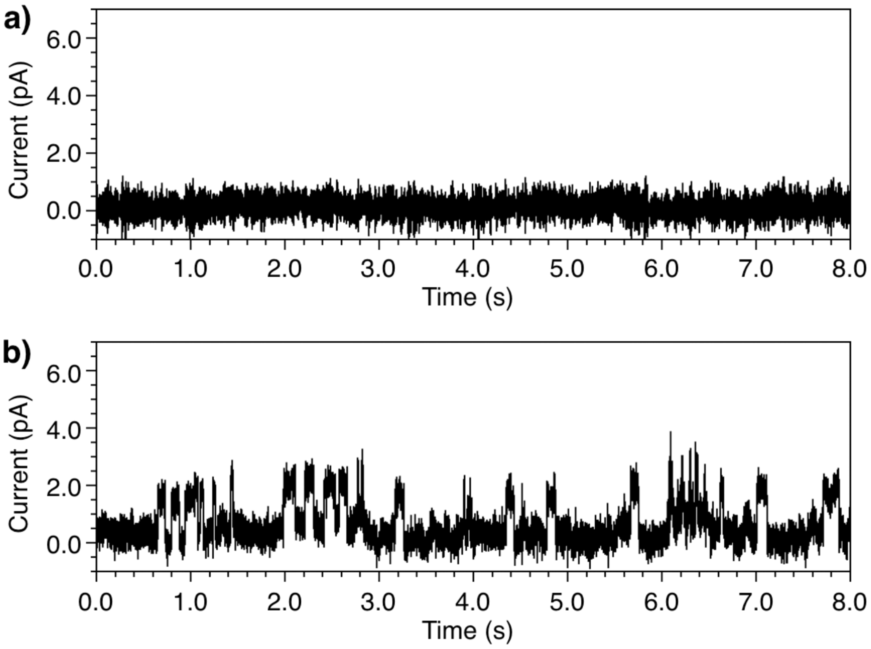

Supplementary Fig. 23 | Current traces of DOPC•2mer BLM (post-loading) upon addition of PA.

Current traces of a DOPC•**2mer**^{post} BLM in HEPES buffer after addition of PA into the a) lower chamber followed by b) the upper chamber. [DOPC]/[**2mer**] = 60000/1. [PA] = 200 nM. Buffer: 20 mM HEPES, 50 mM KCl, 2.0 mM $MgCl_2$, pH 7.5. Applied voltage: 100 mV.

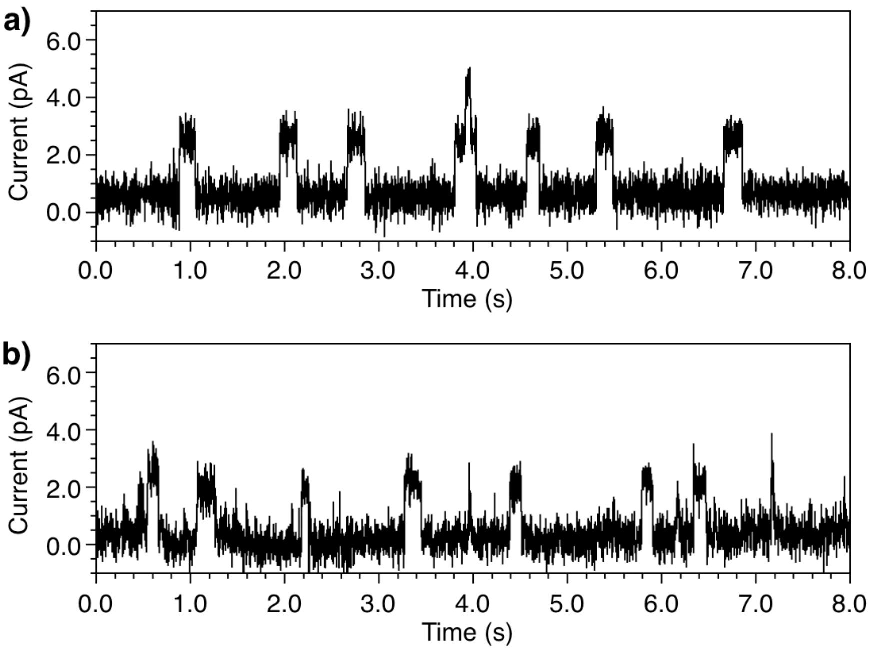

Supplementary Fig. 24 | Current traces of DOPC•2mer BLM (pre-loading) upon addition of PA.

Current traces of a DOPC•**2mer**^{pre} BLM in HEPES buffer after addition of PA into the a) upper chamber or b) lower chamber. The BLM was formed horizontally at the orifice by painting the *n*-decane solution of a mixture of DOPC and **2mer**. [DOPC]/[**2mer**] = 60000/1. [PA] = 200 nM. Buffer: 20 mM HEPES, 50 mM KCl, 2.0 mM $MgCl_2$, pH 7.5. Applied voltage: 100 mV.

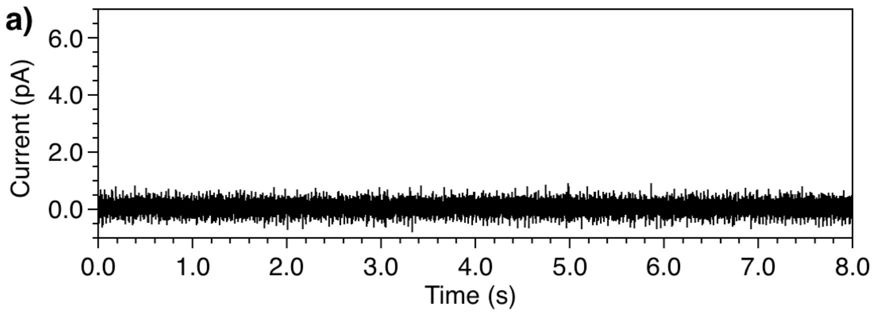

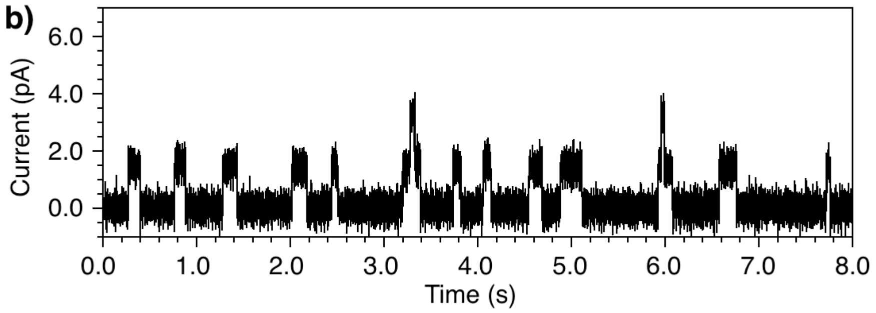

Supplementary Fig. 25 | Current traces of DOPC•2mer BLM (post-loading) upon dilution and re-addition of PA.

Current traces of a DOPC•**2mer**^{post} BLM in HEPES buffer a) after addition of PA (200 nM) into the upper chamber followed by 100-times dilution, and b) subsequent addition of PA (200 nM) into the upper chamber. [DOPC]/[**2mer**] = 60000/1. [PA] = 200 nM. Buffer: 20 mM HEPES, 50 mM KCl, 2.0 mM $MgCl_2$, pH 7.5. Applied voltage: 100 mV.

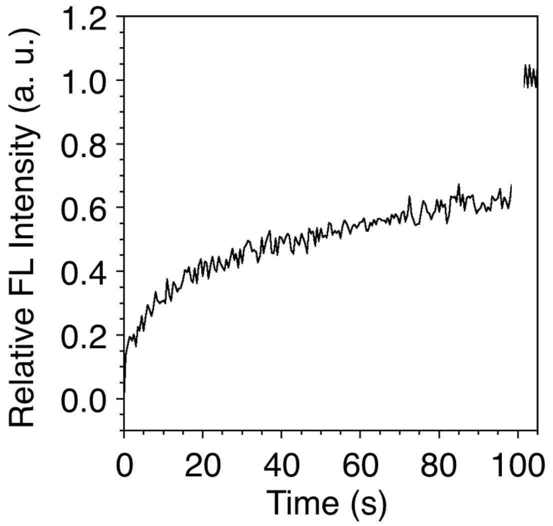

Supplementary Fig. 26 | HPTS assay of DOPC•2mer LUVs (post-loading) upon addition of $Ca(OH)_2$

Changes in 510-nm fluorescence intensity of HPTS encapsulated in DOPC•**2mer**^{post} LUVs in HEPES buffer containing PA at 20 °C as a function of time after the addition of $Ca(OH)_2$ at 0 sec followed by addition of 1.0 wt% triton X-100 at 100 sec ([DOPC] = 200 μM, [**2mer**] = 10 μM, [PA] = 20 mM, [HPTS] = 30 μM, 20 mM HEPES, 50 mM KCl, pH 7.1, excitation at λ = 460 nm, emission at 510 nm). ΔpH = 0.8 (7.1 to 7.9).

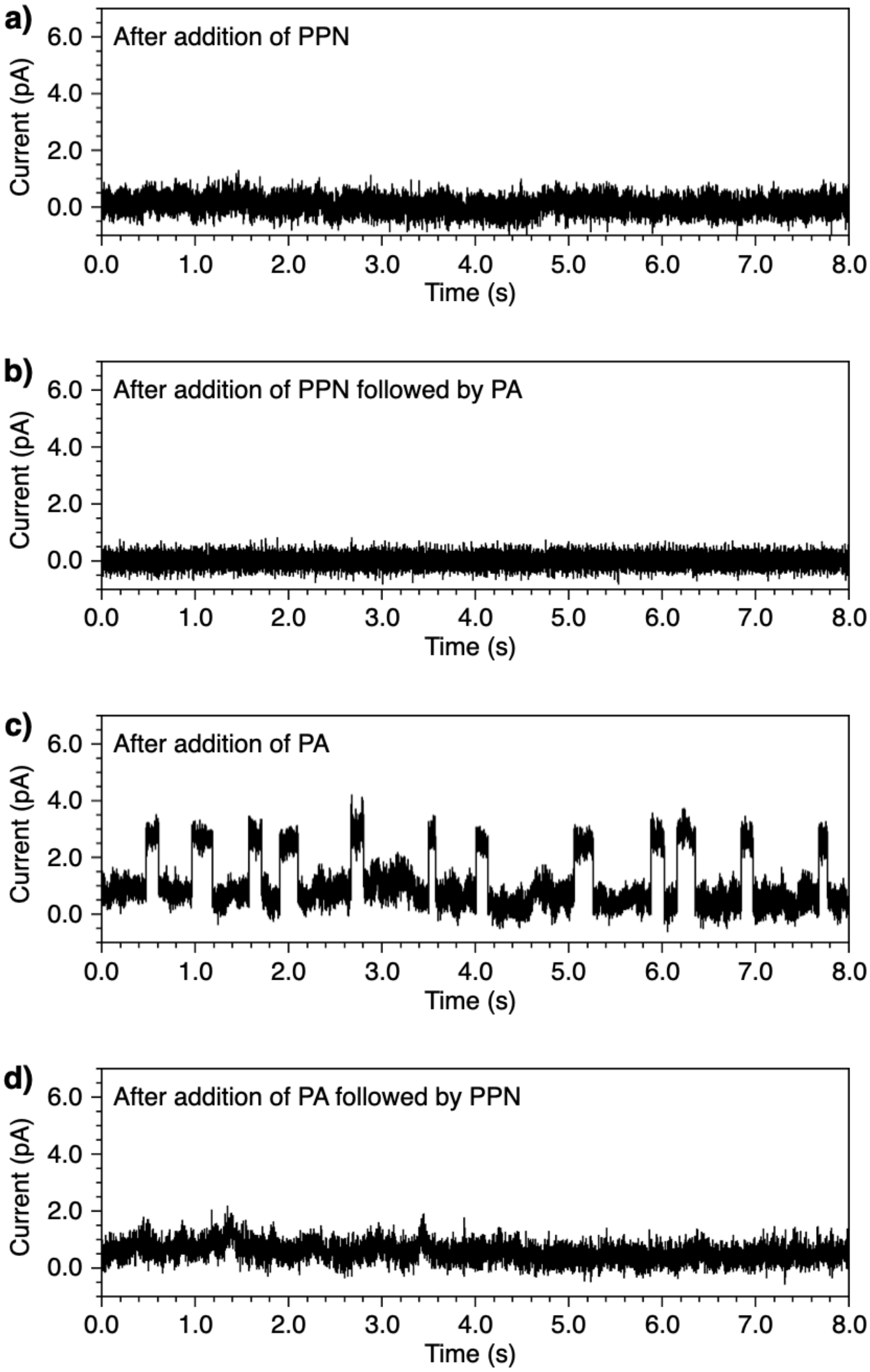

Supplementary Fig. 27 | Current traces of DOPC•2mer BLM (post-loading) upon addition of PA and PPN.

Current traces of a DOPC•**2mer**^{post} BLM in HEPES buffer after addition of a) PPN, b) PPN followed by PA, c) PA and d) PA followed by PPN in the upper chamber at 20 °C. [DOPC]/[**2mer**] = 60000/1. Final concentrations of PA and PPN in the upper chamber are 200 nM and 50 nM, respectively. Buffer: 20 mM HEPES, 50 mM KCl, 2.0 mM $MgCl_2$, pH 7.5. Applied voltage: 100 mV.

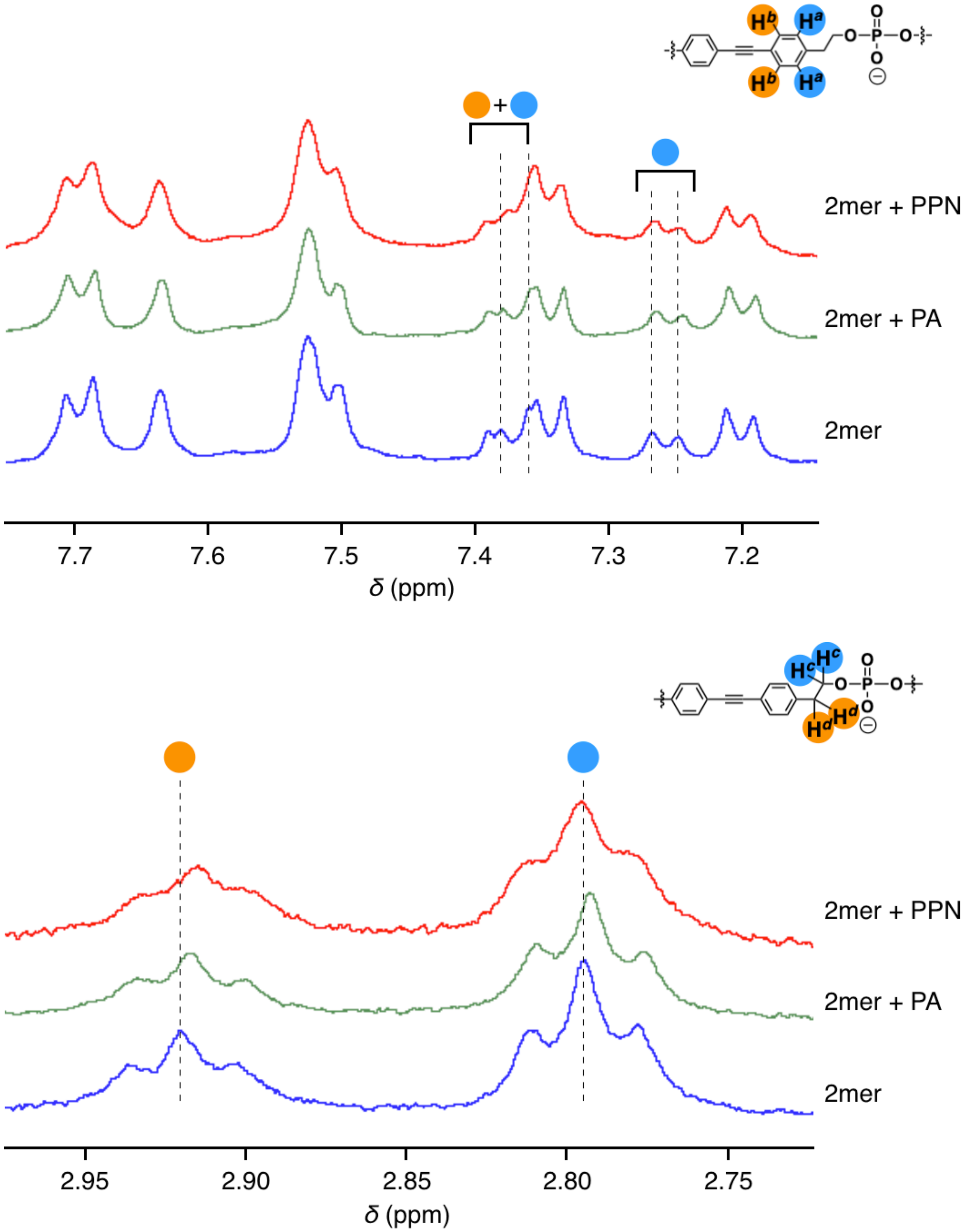

Supplementary Fig. 28 | ^{1}H NMR spectra of 2mer in the presence of PA and PPN.
^{1}H NMR spectra of **2mer** in $CDCl_3$ in the absence (blue) and presence of PA (green) and PPN (red) at 25 °C. [PA]/[**2mer**] = [PPN]/[**2mer**] = 1.0.

In the aromatic region (upside), H^a and H^b protons of **2mer** indicate two signals at 7.26 and 7.38 ppm (7.26 ppm: H^a, 7.38 ppm: H^a and H^b overlapped). Upon addition of PA to **2mer**, the signal at 7.26 ppm (H^a) showed slight upfield shift, while the signal at 7.38 ppm (H^a and H^b) hardly shifted. Addition of PPN to **2mer** resulted in upfield shifts of the signals at both 7.26 and 7.38 ppm. Hence, it is suggested that PA is located near one of the H^a protons and PPN stacks on the phenylene ring bearing Ha and H^b protons of **2mer**. In the aliphatic region (downside), H^c and H^d protons in the ethylene groups between the phosphate and aromatic units of **2mer** indicate two signals at 2.79 and 2.92 ppm (2.79 ppm: H^c, 2.92 ppm: H^d). Upon addition of PA to **2mer**, both of the aliphatic signals (H^c and H^d) showed upfield shifts. Meanwhile, only H^d signal shifted upon addition of PPN to **2mer**. Thus, it is suggested that PA is bound at the rim of the channel of **2mer** near the phosphate groups through the electrostatic interactions, whereas PPN is inserted into the aromatic cavity likely by the hydrophobic interaction.

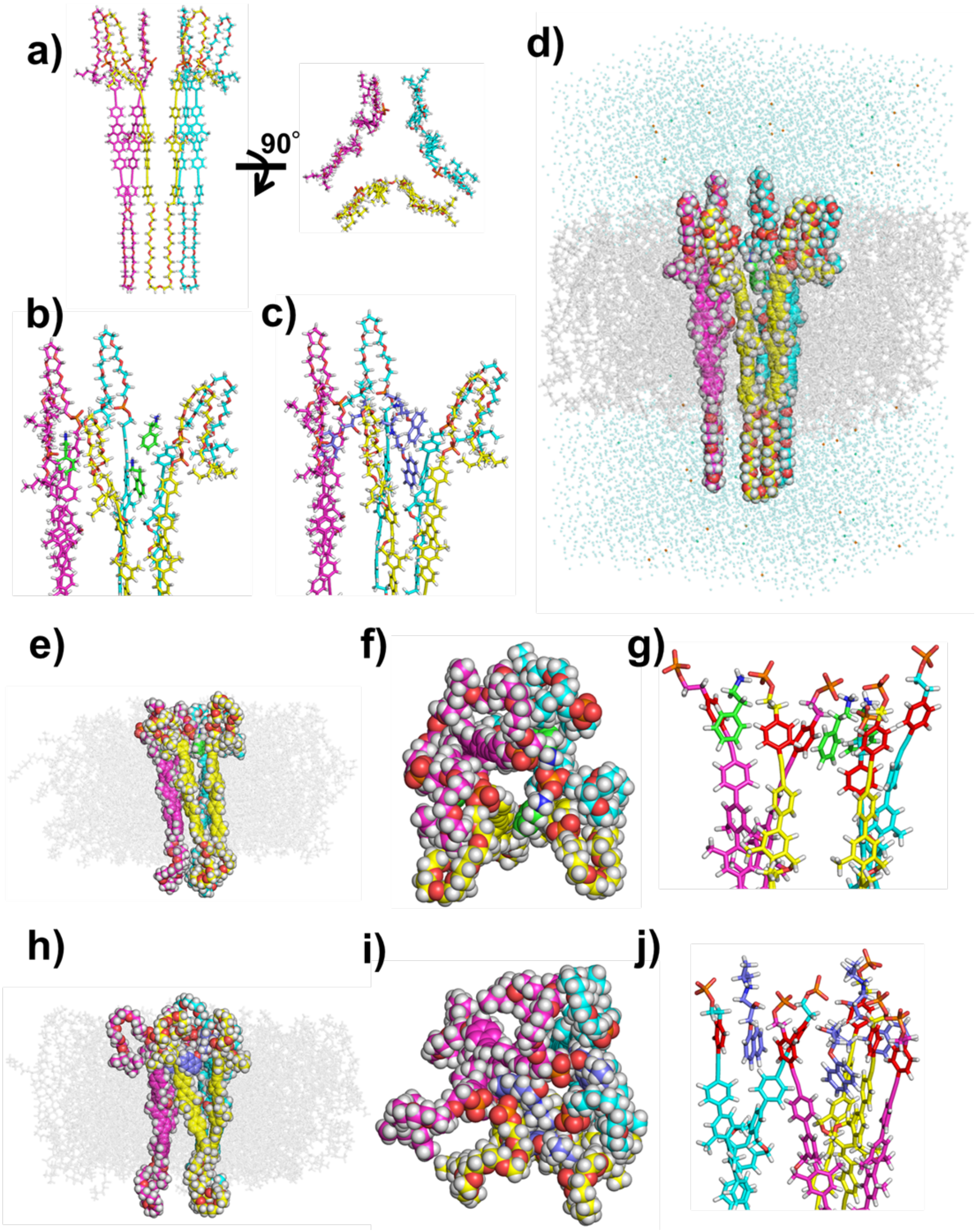

Supplementary Fig. 29 | Modelling of 2mer and ligand complexes.

Initial three-dimensional structure model of a) three **2mer** colored yellow, cyan, and magenta, b) a complex of three-**2mer** and three PA colored green, and c) a complex of three-**2mer** and three PPN colored purple. In b) and c), only the ligand binding part is shown. d) An all-atom model of the three-**2mer** and PA complex (sphere representations) embedded in DOPC

lipid bilayer (gray sticks) and water molecules (cyan dots) used in molecular dynamics simulations as an initial structure. A structural model after a 500 ps MD simulation of three-**2mer** and e–g) PA complex or h–j) PPN complex. A side view of the complex (colored spheres) for e) PA or h) PPN and membrane (gray sticks) is shown. The complex with f) PA or i) PPN is viewed from the ligand binding part. The ligand binding part for the complex with g) PA or j) PPN is enlarged. For easy visualization, the oligoEG part is not shown, and benzene adjacent to the phosphate group in **2mer** is colored red.

Modellings of the **2mer** and PA or PPN complex were conducted using all-atom molecular dynamics simulations in which **2mer**, ligand, DOPC membrane, ion, and water molecules were included at atomic resolution. According to structural insights suggested by experimental results, a three-**2mer** complex was modeled (Supplementary Fig. 29a), and PA (Supplementary Fig. 29b) or PPN (Supplementary Fig. 29c) was located at each **2mer**. In order to survey structural features of the complex with different ligands, the structure of the complex was equilibrated in DOPC bilayer membrane and water molecules (Supplementary Fig. 29d), and a structural model was obtained after a 500 ps simulation.

The complexes with ligands kept a three-**2mer** complex (Supplementary Fig. 29e,h), and however, the pore size among three **2mer** differed for the PA and PPN bound complexes. The pore size of the PA bound complex (Supplementary Fig. 29f) was larger than the PPN bound complex (Supplementary Fig. 29i), and PPN filled the pore among the three-**2mer** complex. This structural feature is consistent with the experimental suggestions of the ion permeability, depending on PA or PPN binding.

The structural difference of the complex with different ligands seems to be originated from the different interaction patterns between ligands and **2mer**: PA mainly interacted with the phosphate group of **2mer** through the electrostatic interactions. In contrast, PPN mainly formed hydrophobic packings with aromatic groups of **2mer**, and the binding region of **2mer** with PPN was much larger than that of PA, reflecting that the molecular size of PPN is larger than that of PA. In addition, PA were bound at the rim of the **2mer** complex, and PPN was located at the deeper position of the pore than that of PA, despite the fact that PA and PPN were set to the same depth at the initial structure. These structural insights are consistent with the experimental findings by NMR studies (Supplementary Fig. 28).

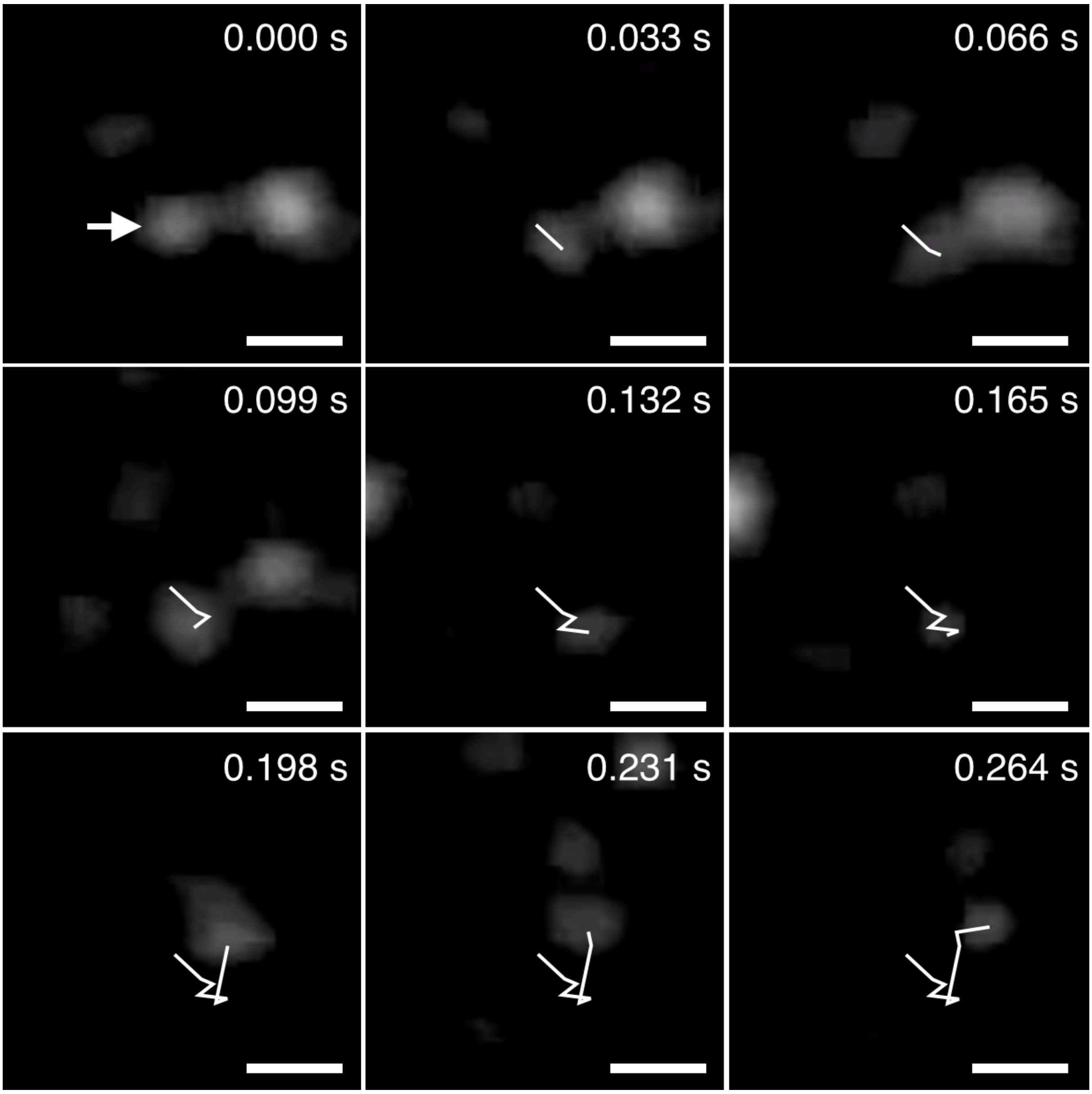

Supplementary Fig. 30 | TIRF microscopic observation of Cy3-2mer added to L cells.

Snapshots of total internal reflection fluorescence microscopic observation of **Cy3-2mer** added to L cells at an interval of 0.05 s at 25 °C. White lines represent the track of lateral migration of the fluorescence spot pointed by a white arrow. Scale bars: 1.0 μm.

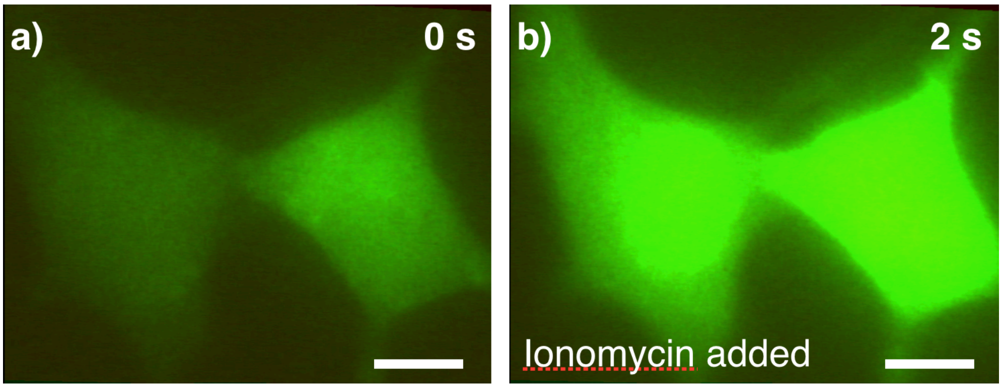

Supplementary Fig. 31 | Fluorescence microscopic observation of L cells upon addition of ionomycin.

Snapshots of fluorescence microscopic observation of L cells encapsulating Fluo-4 at 25 °C at a) 0 s (at the beginning of the observation) and b) 2 s (after the addition of ionomycin). Scale bars: 5.0 μm.

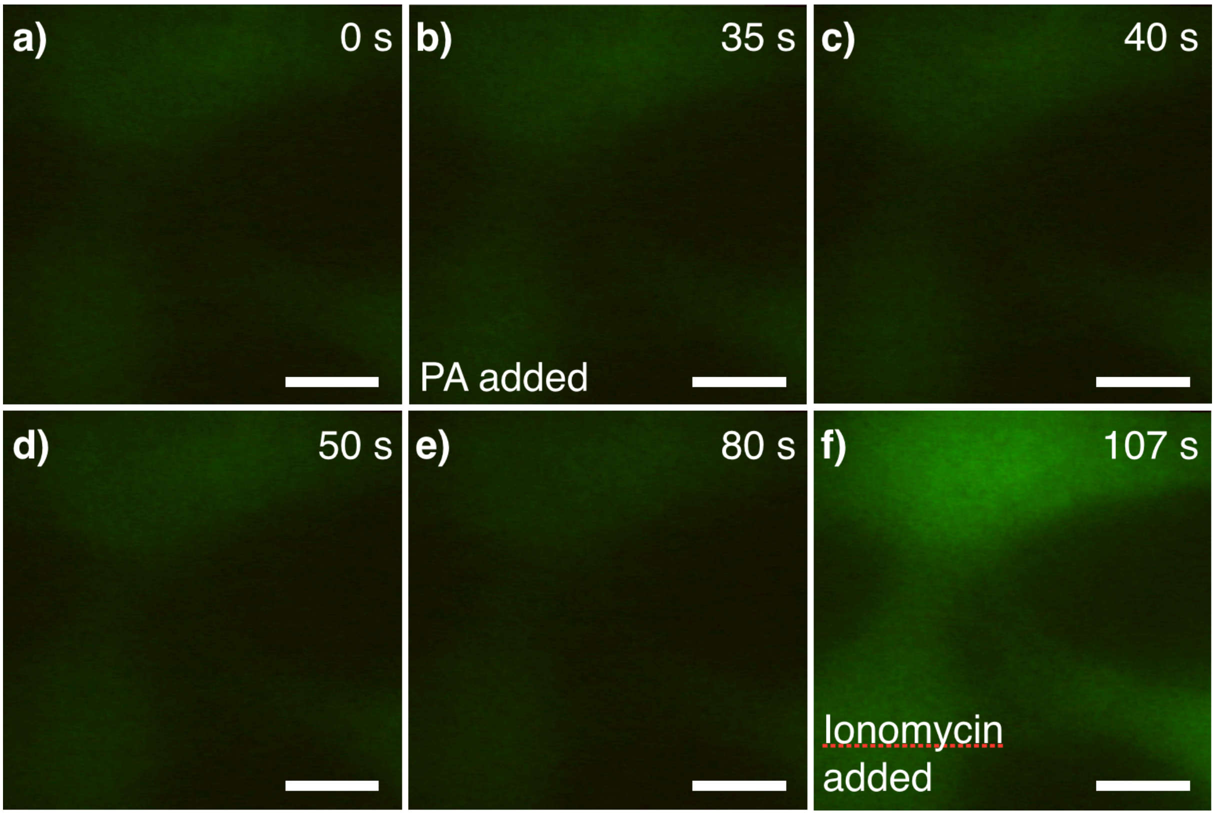

Supplementary Fig. 32 | Fluorescence microscopic observation of L cells upon addition of PA without 2mer.

Snapshots of fluorescence microscopic observation of L cells encapsulating Fluo-4 at 25 °C at a) 0 s (at the beginning of the observation), b–e) 35–80 s (after the addition of PA) and f) 107 s (after the addition of ionomycin as a positive control). Scale bars: 5.0 μm.

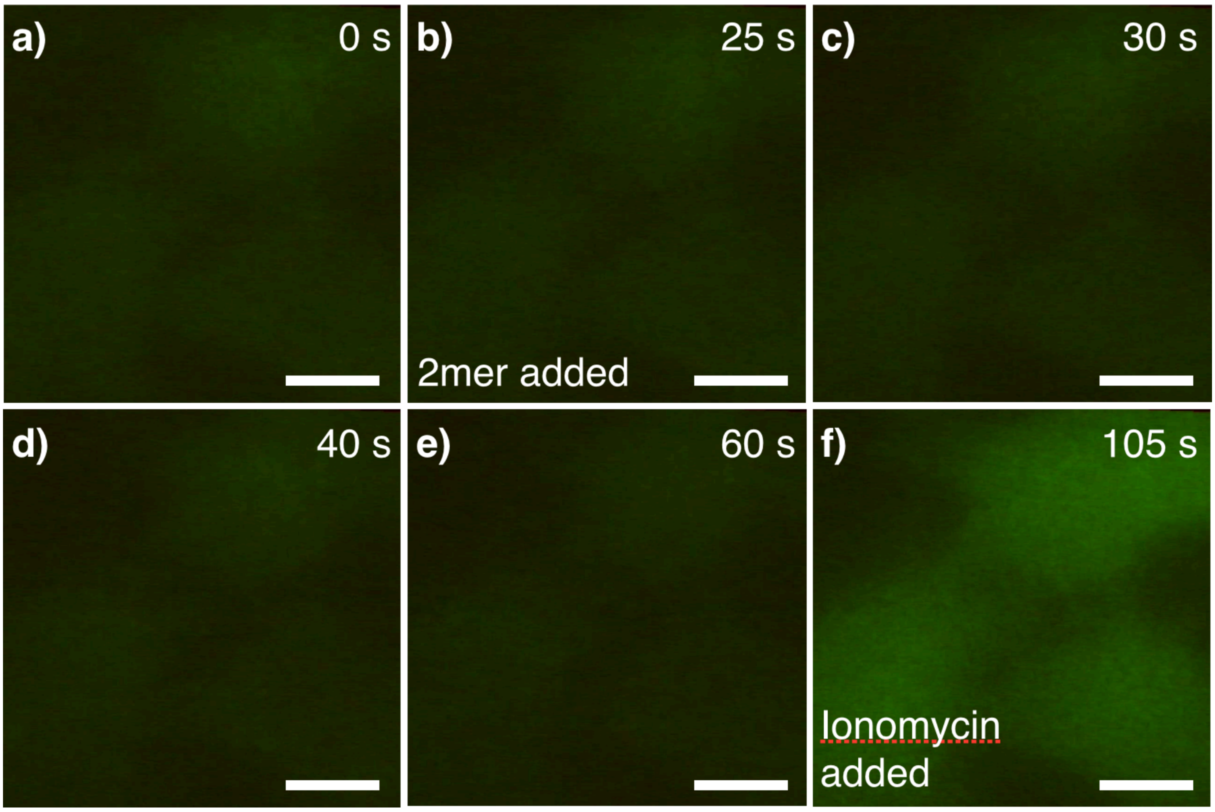

Supplementary Fig. 33 | Fluorescence microscopic observation of L cells upon addition of 2mer.

Snapshots of fluorescence microscopic observation of L cells encapsulating Fluo-4 at 25 °C at a) 0 s (at the beginning of the observation), b–e) 25–60 s (after the addition of **2mer**) and f) 105 s (after the addition of ionomycin as a positive control). Scale bars: 5.0 μm.

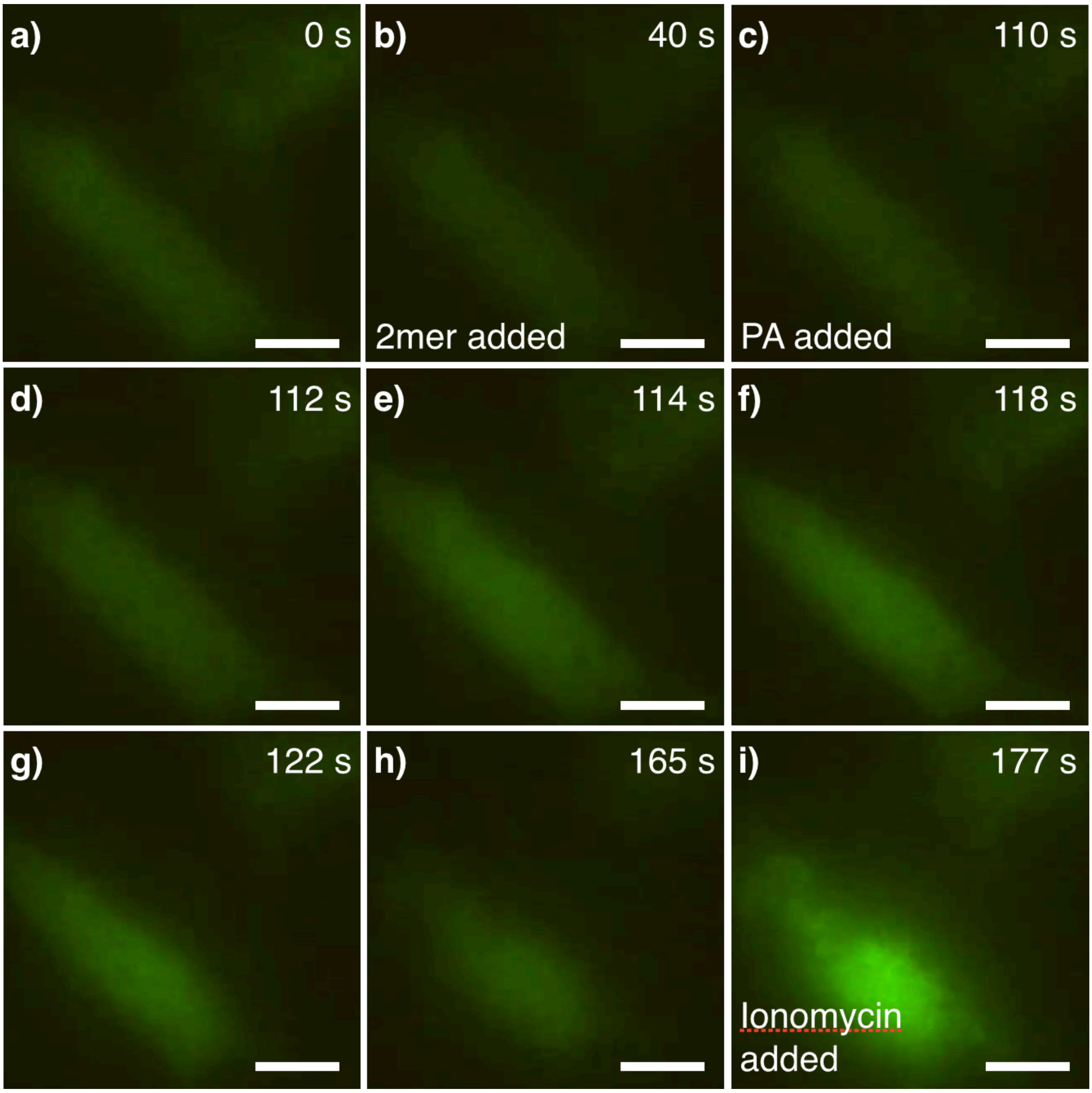

Supplementary Fig. 34 | Fluorescence microscopic observation of L cells upon addition of 2mer and PA.

Snapshots of fluorescence microscopic observation of L cells encapsulating Fluo-4 at 25 °C at a) 0 s (at the beginning of the observation), b) 40 s (after the addition of **2mer**), c–h) 110–165 s (after the addition of PA), and i) 177 s (after the addition of ionomycin as a positive control). Scale bars: 5.0 µm.

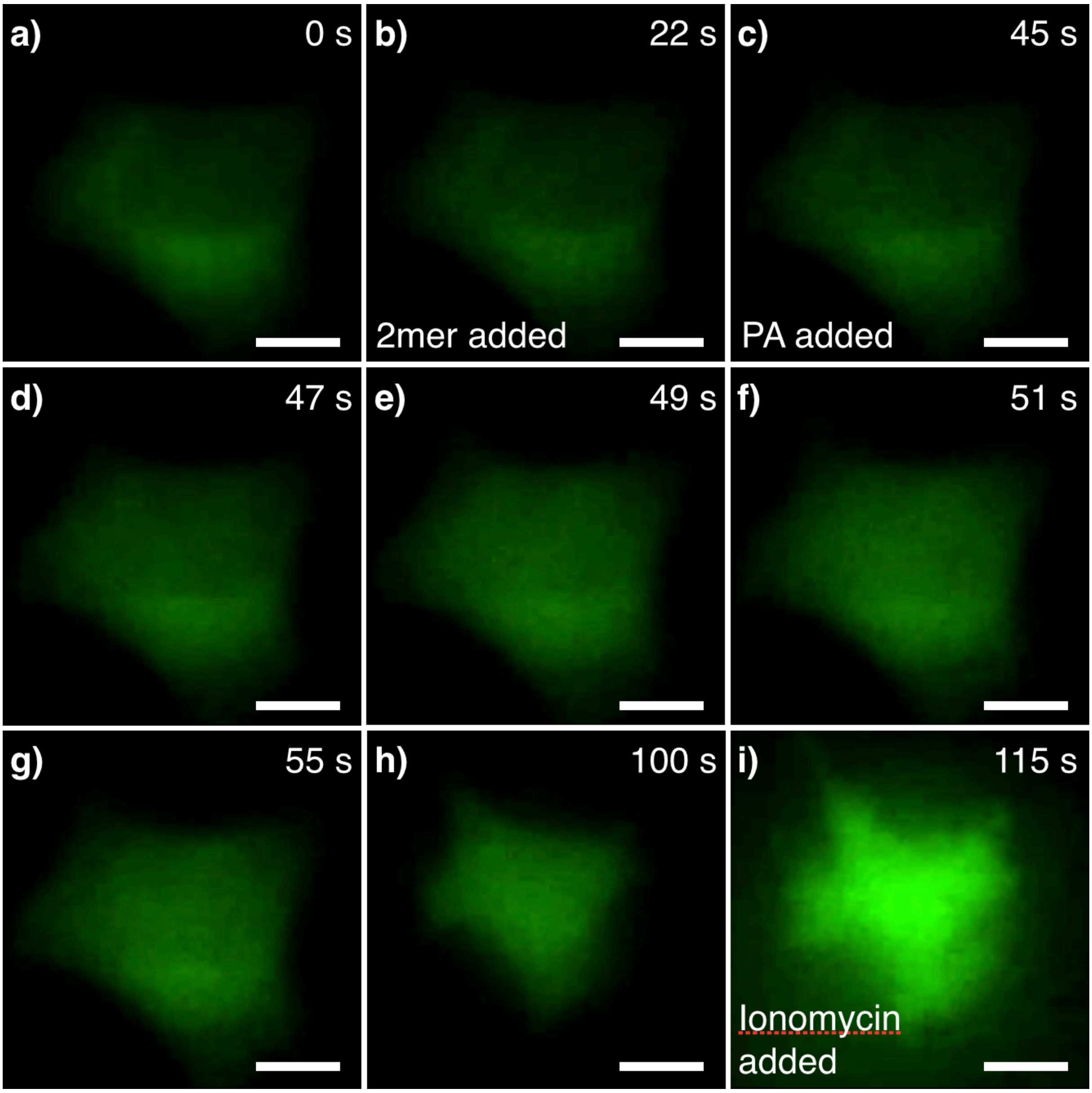

Supplementary Fig. 35 | Fluorescence microscopic observation of L cells upon addition of 2mer and PA.

Snapshots of fluorescence microscopic observation of L cells encapsulating Fluo-4 at 25 °C at a) 0 s (at the beginning of the observation), b) 22 s (after the addition of **2mer**), c–h) 45–100 s (after the addition of PA), and i) 115 s (after the addition of ionomycin as a positive control). a–h) correspond to Figure 6a–6h. Scale bars: 5.0 µm.

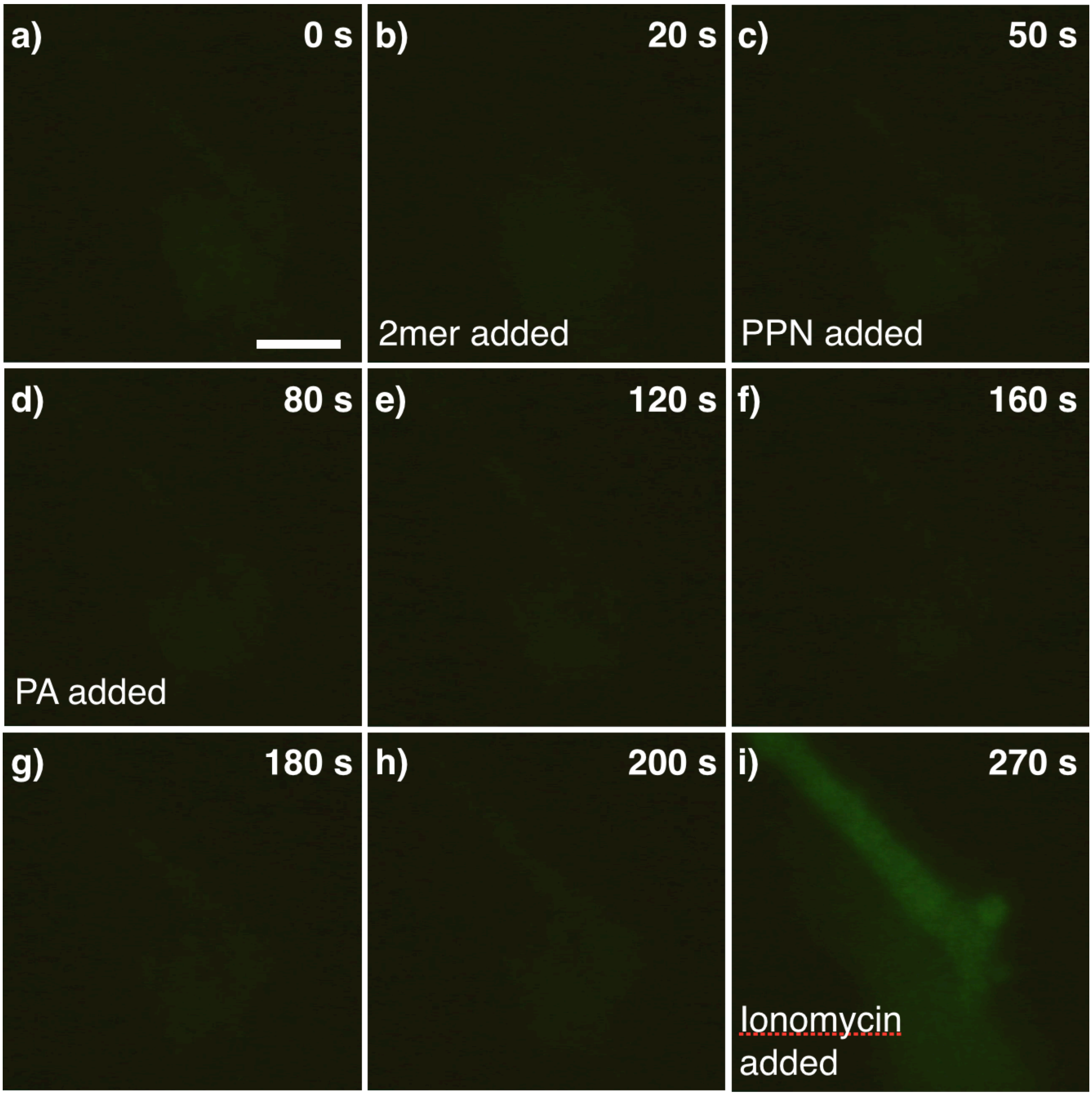

Supplementary Fig. 36 | Fluorescence microscopic observation of L cells upon addition of 2mer, PPN and PA.

Snapshots of fluorescence microscopic observation of L cells encapsulating Fluo-4 at 25 °C at a) 0 s (at the beginning of the observation), b) 20 s (after the addition of **2mer**), c) 50 s (after the addition of PPN), (d–h) 80–200 s (after the addition of PA), and i) 270 s (after the addition of ionomycin as a positive control). Scale bars: 5.0 µm.

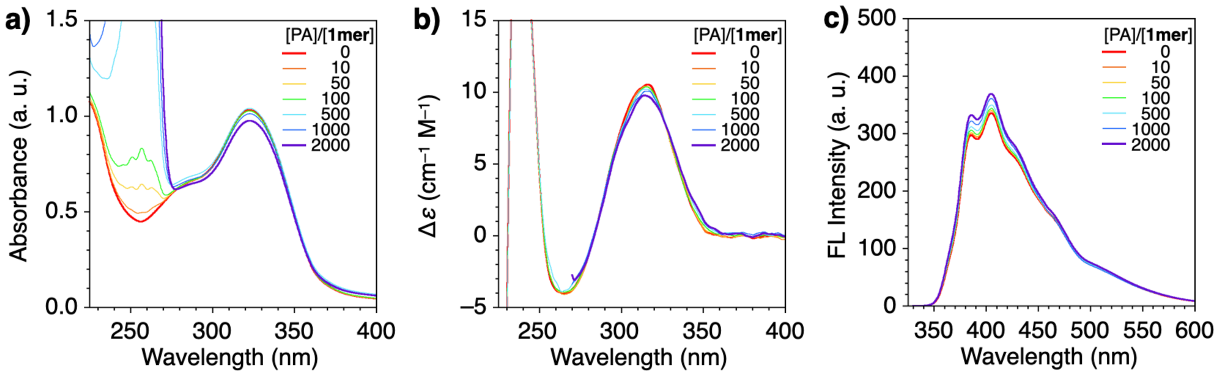

Supplementary Fig. 37 | UV-vis absorption, CD and fluorescence spectral studies of DOPC•1mer LUVs (pre-loading) upon addition of PA.

a) UV-vis absorption, b) CD and c) fluorescence spectral changes of DOPC•**1mer** LUVs ([DOPC] = 200 μM, [**1mer**] = 20 μM) in HEPES buffer at 20 °C upon titration with PA at [PA]/[**1mer**] = 0.0, 10, 50, 100, 500, 1000 and 2000. Excitation at λ = 325 nm. In b), partial spectral curve at [PA]/[**1mer**] = 2000 (λ < 270 nm) is eliminated due to unreliable signals caused by the strong absorption by PA. The LUVs were prepared by evaporation of a $CHCl_3$ solution of a mixture of DOPC and **1mer** followed by hydration and extrusion (pre-loading method).

Supplementary Table

THF-d_8/D_2O	Diffusion constant (10^{10} m^2 s^{-1})	Hydrodynamic diameter (nm)
100/0	3.3	1.4
90/10	2.5	0.97
75/25	1.7	0.99
50/50	1.3	1.1

Supplementary Table 1 | Diffusion constants and hydrodynamic diameters of 2mer.

Diffusion constants and hydrodynamic diameters of **2mer** in a mixture of THF-d_8 and D_2O at 20 °C evaluated by DOSY measurements.

Supplementary References

1. Montoya-Pelaez, P. J., Uh, Y.-S., Lata, C., Thompson, M. P., Lemieux, R. P. & Crudden, C. M. The synthesis and resolution of 2,2'-, 4,4'-, and 6,6'-substituted chiral biphenyl derivatives for application in the preparation of chiral materials. *J. Org. Chem.* **71**, 5921–5929 (2006).
2. Thompson, M. P. & Lemieux, R. P. Chiral induction in nematic and smectic C liquid crystal phases by dopants with axially chiral 1,11-dimethyl-5,7-dihydrodibenz[*c*,*e*]thiepin cores. *J. Mater. Chem.* **17**, 5068–5076 (2007).
3. Lakowicz, J. R. *Principles of Fluorescence Spectroscopy, 3rd ed.* (Springer, 2006).
4. Muraoka, T., Endo, T., Tabata, K. V., Noji, H., Nagatoishi, S., Tsumoto, K., Li, R. & Kinbara, K. Reversible ion transportation switch by a ligand-gated synthetic supramolecular ion channel. *J. Am. Chem. Soc.* **136**, 15584–15595 (2014).
