# Supplementary material for: A synthetic ion channel with anisotropic ligand response
Source: Nat Commun. 2020 Jun 10;11:2924. doi: 10.1038/s41467-020-16770-z (PMC7287108; doi:10.1038/s41467-020-16770-z)
Supplement: Supplementary file 2 — Description of Additional Supplementary Files [file 41467_2020_16770_MOESM2_ESM.docx]

Description of Additional Supplementary Files

**Title: Supplementary Movie 1** | Localization of multiblock amphiphile in plasma membrane.

Total internal reflection fluorescence microscopic observation of Cy3-2mer in the plasma membrane

of mouse L cell at 25 °C. Scale bar: 1.0 µm.

**Title: Supplementary Movie 2** | Addition of Cy3 fragment to an aqueous medium containing cells.

Total internal reflection fluorescence microscopic observation of Cy3 fragment added to an aqueous

medium containing mouse L cell at 25 °C. Scale bar: 1.0 µm.

**Title: Supplementary Movie 3** | Ligand-gated ion transportation of multiblock amphiphile embedded in plasma membrane.

Fluorescence microscopic observation of L cells encapsulating Fluo-4 at 25 °C upon addition of 2mer,

PA followed by ionomycin. Excitation at 488 nm. Scale bars: 5.0 μm. 3-Times fast video rate.
